# Reduced Models of Cardiomyocytes Excitability: Comparing Karma and FitzHugh–Nagumo

**DOI:** 10.1007/s11538-021-00898-0

**Published:** 2021-07-02

**Authors:** Maria Elena Gonzalez Herrero, Christian Kuehn, Krasimira Tsaneva-Atanasova

**Affiliations:** 1 Department of Mathematics, Technical University Munich, Bolzmannstr. 3, 85748 Garching, Germany; 2 Department of Mathematics, College of Engineering, Mathematics and Physical Sciences, University of Exeter, Exeter, EX4 4QJ UK; 3 Institute for Advanced Study, Technical University of Munich, Lichtenbergstrasse 2 a, 85748 Garching, Germany

**Keywords:** Cardiac cells, Mathematical modelling, Fast–slow systems, Singular perturbation, Travelling waves

## Abstract

Since Noble adapted in 1962 the model of Hodgkin and Huxley to fit Purkinje fibres, the refinement of models for cardiomyocytes has continued. Most of these models are high-dimensional systems of coupled equations so that the possible mathematical analysis is quite limited, even numerically. This has inspired the development of reduced, phenomenological models that preserve qualitatively the main feature of cardiomyocyte’s dynamics. In this paper, we present a systematic comparison of the dynamics between two notable low-dimensional models, the FitzHugh–Nagumo model (FitzHugh in Bull Math Biophys 17:257–269, 1955, J Gen Physiol 43:867–896, 1960, Biophys J 1:445–466, 1961) as a prototype of excitable behaviour and a polynomial version of the Karma model (Karma in Phys Rev Lett 71(7):16, 1993, Chaos 4:461, 1994) which is specifically developed to fit cardiomyocyte’s behaviour well. We start by introducing the models and considering their pure ODE versions. We analyse the ODEs employing the main ideas and steps used in the setting of geometric singular perturbation theory. Next, we turn to the spatially extended models, where we focus on travelling wave solutions in 1D. Finally, we perform numerical simulations of the 1D PDE Karma model varying model parameters in order to systematically investigate the impact on wave propagation velocity and shape. In summary, our study provides a reference regarding key similarities as well as key differences of the two models.

## Introduction

Excitability is a fundamental property of cardiomyocytes that defines their ability to propagate electrical activity. This electrical activity is coupled to cardiac contractility that controls the heart’s beat and hence is critical for healthy functioning of the heart (Bers 2002). Abnormalities in excitation–contraction coupling mechanisms are often associated with cardiac disfunction, such as arrhythmia (Chakrabarti and Stuart 2005). This has motivated the exploration of different methodologies for exploiting enhanced understanding of cardiomyocytes excitability that could potentially lead to improvement in clinical outcomes. A representative example that has used this opportunity is the advanced high-throughput pharmaceutical screening based on primary cardiac myocytes (Meyer et al. 2004).

The organised flow of ionic currents across cardiomyocyte’s cell membrane controls cardiac excitability. Hodgkin and Huxley (1952) proposed the first ionic model to represent excitable behaviour, namely action potentials in a nerve fibre. This model is given by a 4-dimensional system of differential equations with one variable for the voltage and three gating variables for the ion channels. By adapting those equations to cardiac cells Noble (1962) developed a similar model for Purkinje cells opening up a new line in mathematical modelling focused on the heart. Since then there have been many different models either including different currents or ionic pumps, see for example further developments of the Noble model (McAllister et al. 1975; DiFrancesco and Noble 1985), and also models for other parts of the heart instead of Purkinje fibres like, e.g. ventricular cells in the Beeler–Reuter model (Beeler and Reuter 1977). To find a more extensive list of cardiac cell models, see Fenton and Cherry (2008).

All the models mentioned above are quite complex with at least four variables and highly nonlinear. That makes analytical results often impossible, or at least extremely cumbersome. Furthermore, many models are even too complex for detailed numerical analysis and simulation for many parameters. This is why FitzHugh (1955, 1960, 1961) developed a simplified model of the Hodgkin–Huxley equations as a variation of the van der Pol oscillator (van der Pol 1920, 1926), which focused on capturing the excitable properties of the system. Almost at the same time, Nagumo et al. (1962) published the corresponding electrical circuit. Their model reduces the four-dimensional system of Hodgkin and Huxley into two equations separating the fast timescale *t* of the excitation and the slow timescale $\tau$ of recovery. Similarly to neuronal excitability the electrical excitability of the heart could be mathematically modelled using differential equation describing the action potential propagation and in its simplest form via monodomain models (Aliev and Panfilov 1996) that are well suited for a range of questions involving excitability (Potse et al. 2006). With regard to the electrical behaviour, the simplest phenomenological model for excitable cells is the FitzHugh–Nagumo (FHN) model (FitzHugh 1961; Nagumo et al. 1962) and its popular modification for cardiac cells, the Aliev-Panfilov model (Aliev and Panfilov 1996). The equations are given (in the fast timescale *t*) by

1.1 $$\begin{aligned} \begin{aligned} v'&= \frac{\partial v}{\partial t} =D\varDelta v + v-\frac{v^3}{3}-w+I\\ w'&=\frac{\partial w}{\partial t} = \varepsilon (v+a-bw) \end{aligned} \end{aligned}$$

where *v* corresponds to the voltage and *w* to a slow gating variable, *a*, *b* are model parameters, *D* is the diffusion coefficient and *I* is an external current. Although its representation of nerve fibres or cardiac cells is not as precise as it would be with a more complex model, the FitzHugh–Nagumo (FHN) model has been studied extensively in the literature and is one of the prototype models for excitable media due to its simplicity.

In the same spirit as FitzHugh, Karma (1993, 1994) developed a reduced version of the Noble model characterised by similar to the FitzHugh–Nagumo fast–slow structure. Here, we present a systematic analysis of the Karma model comparing it to the FitzHugh–Nagumo model given that both models have been extensively used to model the behaviour of cardiomyocytes. Some additional references can be found in Beck et al. (2008), Mitchell and Schaeffer (2003) for the Karma model and Barkley (1991), Biktashev (2003), Postnikov and Titkova (2016) for the FitzHugh–Nagumo model. In Sect. 2 we show under what assumptions the version of the Karma model introduced in Karma (1993) and Karma (1994) are equivalent. In Sect. 3, we present a systematic comparison of the FitzHugh model (1.1) and the Karma model as defined in Sect. 2. We conclude our comparison in Sect. 4 with numerical simulations of the full PDE systems with a focus on the Karma model.

## The Karma Model

As mentioned above, the Karma model introduced in (Karma 1993) is a two-variable model involving one fast and one slow variable similarly to the FitzHugh–Nagumo model. The important advantages are that Karma ensured additional dynamic features that play an important role for cardiac cells in the Noble model. Namely, he focused on reproducing the insensitivity of the wave-front velocity with respect to the slow variable, obtaining a fixed phase condition determining the position of the wave-back and the presence of alternans for small amplitude wave trains. The corresponding equations read

2.1 $$\begin{aligned} \begin{aligned} \varepsilon \dot{E}&=\varepsilon \frac{\partial E}{\partial \tau } = \varepsilon ^2\varDelta E-E+\left( E^*-\left( \frac{n}{n_B}\right) ^M\right) (1-\tanh (E-3))\frac{E^2}{2},\\ \dot{n}&=\frac{\partial n}{\partial \tau } = \theta (E-1)-n, \end{aligned} \end{aligned}$$

for *E* the electrical potential and *n* a slow variable representing the ion channels with the Heaviside or indicator function $\theta (x) = 0$ for $x \le 0$ and $\theta (x) = 1$ for $x > 0$. The parameter $0<n_B<1$ controls the position of the wave-back and the parameter $M\gg 1$ controls the insensitivity of the excitable wave velocity with respect to the slow gating variable *n*. Furthermore, the constant $E^*=1.5415$ has been fitted such that

2.2 $$\begin{aligned} f_E(E,n_B)=\frac{\partial }{\partial E}f_E(E,n_B)=0 \end{aligned}$$

for some *E* where $f_E$ is the right-hand side of the first equation without the diffusion term.

In a follow-up paper, Karma (1994) formalised the model in a slightly more general way as follows

2.3 $$\begin{aligned} \begin{aligned} E'&= \frac{\partial E}{\partial t} = \gamma \varDelta E+\tau _E^{-1}[-E+\left( E^*-{\mathscr {D}}(n)\right) h(E)],\\ n'&= \frac{\partial n}{\partial t} = \tau _n^{-1}[{\mathscr {R}}(n)\theta (E-1)-(1-\theta (E-1))n]. \end{aligned} \end{aligned}$$

One may view $\tau _E$ and $\tau _n$ as defining the scales for the reaction terms at which *E* and *n*, respectively, evolve. We therefore define $\varepsilon =\tau _E/\tau _n$ as a single parameter separating the timescales. Next, to make sure there are exactly two stable equilibria for *n* fixed (corresponding to the depolarised and polarised states) a common choice (Karma 1993, 1994) for the reaction function *h* is

2.4 $$\begin{aligned} h(E)=(1-\tanh (E-3))\frac{E^2}{2} \end{aligned}$$

and the parameter $E^*$ is kept as defined above. Alternatively, a common suggestion (Karma 1993, 1994) is a function of the form $h(E)=E^2-\delta E^3$ which we will treat in more detail later.

To fully define the model we still have to specify the restitution function ${\mathscr {R}}(n)$ and dispersion function ${\mathscr {D}}(n)$. The former is responsible for the length *A* of an action potential after a diastolic or rest interval of length *D*. The latter function defines the relation between the dispersion velocity *c* of a pulse with respect to the previous diastolic interval. In theory, both functions can be chosen to fit arbitrary restitution and dispersion curves of the system to be modelled.

For $\varepsilon$ small, Karma presents the unique relation

2.5 $$\begin{aligned} {\mathscr {R}}(n)=\frac{n}{\left( \frac{\text{ d }A}{\text{ d }D}\right) _{D=\tau _n\ln (1/n)}} \end{aligned}$$

where *A*(*D*) is the restitution curve. Choosing

2.6 $$\begin{aligned} {\mathscr {R}}(n)=\frac{1-(1-e^{-\mathrm{Re}})n}{1-e^{-\mathrm{Re}}} \end{aligned}$$

leads to the restitution curve $A(D)=A_{\mathrm{max}}+\tau _n\ln (1-(1-e^{-\mathrm{Re}})e^{-D/\tau _n})$ and the control parameter Re for the restitution properties.

Similarly, for the dispersion curve with $\varepsilon$ small we have the relation

2.7 $$\begin{aligned} c(D)=\left( \frac{\gamma }{\tau _E}\right) ^{1/2}C({\mathscr {D}}(e^{-D/\tau _n})) \end{aligned}$$

where *c*(*D*) is the dispersion curve and *C* is a function that can be fitted numerically by a third-order polynomial. Karma chooses the simple dispersion function

2.8 $$\begin{aligned} {\mathscr {D}}(n)=n^M \end{aligned}$$

with the control parameter *M* for the dispersion properties.

Having the full definition of the model of 1994, we have to check that both versions of the model, introduced in 1993 and 1994, respectively, are in fact equivalent.

### Proposition 1

The models (2.1) and (2.3) with the functions $h(E),{\mathscr {R}}(n)$ and ${\mathscr {D}}(n)$ chosen as above are equivalent for an appropriate value of $\gamma$.

### Proof

Since the model (2.3), in contrast to (2.1), is written in the fast timescale we start by rescaling time in equation (2.3) with $\tau =\tau _n^{-1}t$

2.9 $$\begin{aligned} \begin{aligned} \varepsilon \dot{E}&=\varepsilon \frac{\partial E}{\partial \tau } = \tau _E\gamma \varDelta E-E+\left( E^*-{\mathscr {D}}(n)\right) h(E)\\ \dot{n}&=\frac{\partial n}{\partial \tau } = {\mathscr {R}}(n)\theta (E-1)-(1-\theta (E-1))n. \end{aligned} \end{aligned}$$

Note that since the model is non-dimensional we can assume without loss of generality $\tau _E=1$. Furthermore, we rescale the gating variable ${{\tilde{n}}}=n/n_B$ and use the parameter transformation $n_B=1-e^{-\mathrm{Re}}$. After dropping the tildes, we have

2.10 $$\begin{aligned} \begin{aligned} \varepsilon \dot{E}&= \tau _E\gamma \varDelta E-E+\left( E^*-\left( \frac{n}{n_B}\right) ^M\right) h(E)\\ \dot{n}&= \theta (E-1)-n \end{aligned} \end{aligned}$$

which differs from the 1993 model (2.1) only in the diffusion parameter. Given that $\tau _E$ is independent of $\varepsilon$ we have again a slightly more general formulation of the same model. By choosing $\gamma =\varepsilon ^2$, we obtain the 1993 model. $\square$

In the remainder of this paper, we use the simpler form of the reaction function mentioned above. To stay as close to the function used by Karma as possible, we have chosen the reaction function

**Fig. 1:**
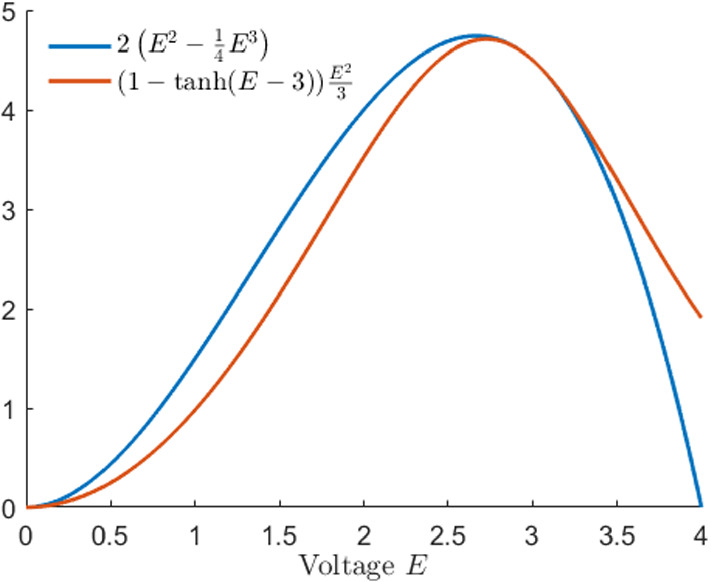
Reaction function (2.4) used by Karma (1993, 1994) (orange) together with the polynomial reaction function (2.11) we are going to analyse in this paper (blue) (Colour figure online)

2.11 $$\begin{aligned} h(E)=2\left( E^2-\frac{1}{4}E^3\right) \end{aligned}$$

which is the third-order Taylor expansion of (2.4) at $E=3$ as shown in Fig. 1.

Due to the change in the reaction function, we have to adapt the parameter $E^*$ such that condition (2.2) is still satisfied, which yields $E^*=1.5$.

Furthermore we notice that the function $\theta$ in the second equation is not continuous. In the Karma model the variable *n* represents a gating variable controlling the opening and closing of specific ion channels on the cell membrane. At rest this channels are closed, however, when the membrane potential exceeds some critical value the channels deform such that ions can flow through. Translating the ion channels’ openning to mathematical equations results in fact in a step function dependent on the potential *E*. Nevertheless, realistically, neither the opening of the channels nor the actual ion flow is instantaneous or perfectly synchronized due to inhomogeneities in the ion distribution, subtle differences in the structure of the channels and many other interacting factors. This is why it is also reasonable to substitute the Heaviside (step) function with some continuous approximation, the most straightforward choice being the piecewise function

2.12 $$\begin{aligned} \theta (x)= {\left\{ \begin{array}{ll} 0 &{}x<0\\ \frac{1}{a}x&{}0\le x\le a\\ 1&{}x>a\\ \end{array}\right. } \end{aligned}$$

for some small constant a. It is clear that this function converges to the Heaviside function as $a\rightarrow 0$ when choosing an appropriate function space. Dynamically very similar and mathematically slightly simpler than (2.12) is the function $\theta (x)$ = max$\{0,x\}$ which has only one point where it is not smooth. Since using either of these choices for q in the model captures equally well the basic properties of excitability as considered by FitzHugh (1961), namely having only one resting state (steady-state equilibrium point) and displaying a threshold phenomenon for a parameter change such as an applied current, in the rest of the paper we will work with the later. It is straightforward to verify that the analysis we perform below can be easily applied to (2.12) with only few minor changes. In any case, it is important to keep in mind that the qualitative properties introduced in (2.5) and (2.7) are only valid for the original choice of q, the Heaviside function, the derivation for any other choice is beyond the scope of this paper and is therefore left for future work.

Summarising, in the reminder of the paper, we are analysing the following model equations

2.13 $$\begin{aligned} \begin{aligned} E'&= D\varDelta E-E+2\left( E^*-n^M\right) (E^2-\delta E^3)\\ n'&= \varepsilon \left( \frac{1}{n_B}\theta (E-1)-n\right) \end{aligned} \end{aligned}$$

with $\theta (x)$ for *E* the potential and *n* a slow gating variable with $E^*=1.5$ and $\delta =0.25$, diffusion coefficient *D* and system parameters $0<n_B<1$ and $M\gg 1$ where we used a mixed form of the scalings in Karma (1993) and Karma (1994).

## FitzHugh–Nagumo and Karma Model

In this section, we proceed analysing and comparing the Karma model (2.13) to the classical FHN system (1.1). We note that both models are two-dimensional systems with a clear fast–slow structure and a diffusive term for the fast variable representing the voltage. A concise introduction into the mathematical theory we will be applying is given in “Appendix A”. In this paper, we are going to focus on illustrating and extracting the main geometric and analytical insights needed in the proofs of different types of dynamics, which makes it more transparent, where the similarities and differences between the two models are, and how to interpret these differences biologically.

### Pure Ordinary Differential Equations (ODE) Models

We start by comparing the simplified version of both models by considering the pure ODE models, i.e. we set the diffusion coefficients equal 0. Hence, we are working with the equations

3.1 $$\begin{aligned} \begin{aligned} E'&=-E+2\left( E^*-n^M\right) (E^2-\delta E^3)+I\\ n'&= \varepsilon \left( \frac{1}{n_B}\theta (E-1)-n\right) \end{aligned} \end{aligned}$$

with $E^*=1.5$, $\delta =0.25$, $M\gg 1$ and $0<n_B<1$ for the Karma model and comparing them to the FHN equations

3.2 $$\begin{aligned} \begin{aligned} v'&=v-\frac{v^3}{3}-w+I\\ w'&=\varepsilon (v+a-bw) \end{aligned} \end{aligned}$$

with $$0<b<1$$ and $$1-\frac{2}{3}b<a<1$$.

#### FitzHugh–Nagumo

The FitzHugh–Nagumo model has been analysed extensively in the literature due to its simplicity and generality. In this paper, we choose the parameter values $a=0.7$ and $b=0.8$ as standard configuration for cardiac cells following FitzHugh (1961) such that for $I=0$ the unique equilibrium is stable corresponding to the polarised state. For completeness, we present now a short overview over the most important steps of the analysis of the ODE system when $I=0$ by exploited the timescale separation in the system. For proofs and deeper analysis of the FitzHugh–Nagumo model, see Jones et al. (1991), Jones (1995), Rauch and Smoller (1978), Rocsoreanu et al. (2000).

**Fig. 2:**
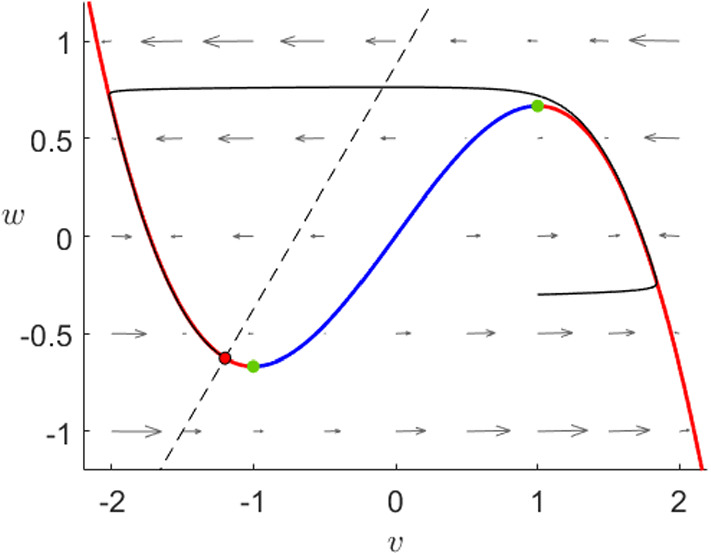
Phase plane of the FitzHugh–Nagumo system (3.2) for $\varepsilon =10^{-2}$ and $I=0$. We can see the critical manifold (3.3) divided by the fold points (green) into two attracting branches (red) and a repelling branch (blue) as well as the *w*-nullcline (dashed) and the unique stable fixed point (black–red). Furthermore, we show a prototypical orbit (black) converging to the global equilibrium (Colour figure online)

In the FitzHugh–Nagumo model (3.2), the flow is always controlled by the third-order critical manifold as we can observe in its phase plane in Fig. 2. The manifold can be divided by its extrema into three branches, where the outer ones are attracting and the middle branch is repelling, therefore the flow away from the critical manifold will approach fast to one of the outer branches. When $I=0$, orbits on the middle or close to the right branch follow the slow flow upwards towards the maximum where they jump fast towards the left branch. Once close to the left branch, every orbit will finally converge towards the sable equilibrium.

The next theorems formalise the main results. The proof is based on the decomposition of the different timescales when $\varepsilon$ is small. For this reason, we first look in Theorem 1 at the singular limit separating the analyse of the layer problem and the reduced system before constructing the candidate orbits. Finally, in Theorem 2 we perturbed the candidate orbits as $\varepsilon >0$ showing that they correspond in fact to solutions of the full FitzHugh–Nagumo model (1.1).

##### Theorem 1

In the singular limit $\varepsilon =0$ of the FitzHugh–Nagumo equations (3.2) with $I=0$, we have a unique stable equilibrium and the third-order critical manifold

3.3 $$\begin{aligned} C_0=\left\{ (v,w):w=v-\frac{v^3}{3}+I\right\} . \end{aligned}$$

Every candidate orbit can be constructed as concatenation of fast segments converging to one of the outer branches of $C_0$ which are attracting and slow segments on the critical manifold. Eventually all orbits converge to the fixed point.

##### Theorem 2

The candidate orbits found in Theorem 1 in the singular limit $\varepsilon =0$ of equations (3.2) when $I=0$ can be perturbed to solution curves of the full system with $\varepsilon >0$.

#### Karma: No External Current

**Fig. 3:**
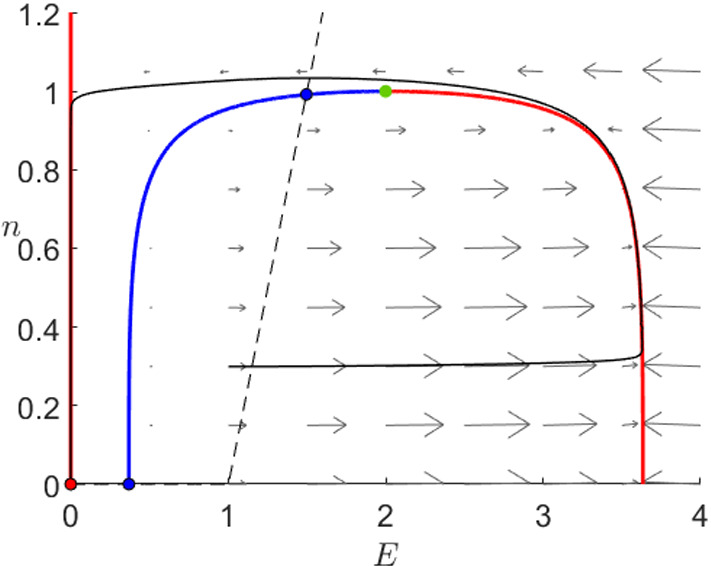
Phase plane of the Karma system (3.1) for $M=4$, $n_B=0.5$, $\varepsilon =10^{-2}$ and $I=0$. We can see the critical manifold (3.4) divided into two attracting branches (red) and a repelling branch (blue), a unique fold point (green) as well as the *n*-nullcline (dashed). Furthermore, we have two unstable equilibria (black–blue) and a unique stable one (black–red) at (0,0) to which a prototypical orbit (black) converges (Colour figure online)

To understand the Karma model equations (3.1), we will now perform a similar analysis. We will show that the dynamics of the Karma model are similar to FitzHugh–Nagumo since again the system is controlled by the critical manifold presenting a similar shape as shown in Fig. 3. As before, we shall indicate the main geometric steps of each proof; see also “Appendix” for more background on the geometric view via geometric singular perturbation theory.

As before, we have two attracting branches separated by a repelling one and exactly one stable equilibrium. An arbitrary orbit will either approach the right branch and then slowly ascend towards the fold point, where it jumps towards the left branch or it approaches directly the left branch where it slowly converges to the stable equilibrium at the origin. In contrast to FHN, the Karma model shows in addition to the stable equilibrium two further unstable fixed points. In general, these points do not affect the overall dynamics, however, the system (3.1) has not only two additional fixed points, which do not converge to the stable equilibrium but also a slow singular heteroclinic orbit between them.

To formally analyse the dynamics, we want to exploit the different timescales as we did for FitzHugh–Nagumo and therefore consider first the limit $\varepsilon =0$ analysing the layer and the reduced problem separately.

##### Remark 1

In contrast to FHN the Karma model is continuous but not smooth due to the rectifier in the second equation. Although some of the analysis techniques used for FitzHugh–Nagumo have to be modified or extended, the existence and uniqueness of solutions are still guaranteed by the Picard–Lindelöff Theorem.

##### Theorem 3

In the singular limit $\varepsilon =0$ of the Karma equations (3.1) with $I=0$, we have a stable, an unstable and a saddle equilibrium and the critical manifold

3.4 $$\begin{aligned} C_0=\left\{ (E,n): E=0 \text { or } n=\root M \of {E^*-\frac{1}{2E(1-\delta E)}}\right\} . \end{aligned}$$

Every candidate orbit can be constructed as concatenation of fast segments converging to one of the outer branches of $C_0$, which are attracting and slow segments on the critical manifold. An orbit will either eventually converge to the stable equilibrium at the origin or it is one of the two unstable fixed points or a unique heteroclinic orbit between them.

##### Proof (Sketch of the Proof)

*Layer problem* Like in FHN, we have the one-dimensional fast subsystem

3.5 $$\begin{aligned} E'=-E+2(E^*-n^M)(E^2-\delta E^3) \end{aligned}$$

where *n* is a parameter. In this subsystem, $E=0$ is always an equilibrium and, depending on *n* we have either two further equilibrium points for $n<1$, exactly one for $n=1$, or no further equilibria when $n>1$. We can calculate the derivative on these points and get

3.6 $$\begin{aligned} J(E;n) =-1+2(E^*-n^M)(2E-3\delta E^2), \end{aligned}$$

which for $(E,n)\in C_0$ is negative on the outer branches, positive in the middle branch and 0 only at

$$\begin{aligned} p_F=(2,1). \end{aligned}$$

Therefore, the critical manifold is normally hyperbolic everywhere except at $p_F$. It is straightforward to check that $p_F$ satisfies all the conditions of a generic fold point.

*Reduced problem* The slow flow has a piece-wise linear nullcline which intersects $C_0$ exactly three times as shown in Fig. 3: twice on the *E*-axis and once with $E,n>0$. Therefore, we have three global fixed points: a stable equilibrium at the origin, a saddle at the intersection of the unstable branch of $C_0$ and the *E*-axis and an unstable fixed point on the unstable branch. The reduced problem is given by

3.7 $$\begin{aligned} \dot{n}=\frac{1}{n_B}\theta (E-1)-n,\quad (E,n)\in C_0 \end{aligned}$$

this means that the slow flow on the left branch as well as on the middle branch below the unstable equilibrium points downwards while it points upwards on the right branch as well as between the unstable node and the fold point $p_F$.

Combining this information, we now want to construct the candidate orbits in the singular limit. Any orbit starting away from the critical manifold will first follow the fast flow converging to one of the attracting branches of $C_0$. The orbits on the right branch follow then the slow flow upwards to $p_F$ where they jump with the fast flow to the left branch. There, all orbits follow the slow flow downwards converging to the global equilibrium (0, 0). Orbits starting on the unstable branch of the critical manifold will either converge to $p_F$ and jump to the left branch if they start above the unstable node or they will converge downwards towards the saddle if they start below the unstable fixed point. $\square$

Finally, we show in the following two theorems that the Karma model for $\varepsilon >0$ has an equivalent behaviour as in the singular limit. To prove this, we want to apply geometric singular perturbation theory (see “Appendix A.2” for more details). Nevertheless, this theory requires differentiability of the system which we loose when $E=1$. Since this line crosses the repelling branch of the critical manifold below the unstable node, we excluded the heteroclinic connection between the unstable fixed points in Theorem 4. This segment will be analysed separately in Theorem 5.

##### Theorem 4

Away from the heteroclinic segment of $C_0$ between the saddle and the unstable node, candidate orbits found in the singular limit $\varepsilon =0$ of equations (3.1) with $I=0$ can be perturbed to solution curves of the full system with $\varepsilon >0$.

##### Proof (Sketch of the Proof)

For this proof, we first need to divide our phase space along the line $E=1$ to be able to guarantee smoothness, therefore we will analyse the left and right parts of the critical manifold separately. Furthermore, Fenichel’s theorems require smooth vector fields defined on ${\mathbb {R}}^2$. In order to satisfy this condition, we extend the systems on each side to the entire real plane so that we will be working with either

3.8 $$\begin{aligned} n' = -\varepsilon n \quad \text {or}\quad n'=\varepsilon \left( \frac{1}{n_B}(E-1)-n\right) \end{aligned}$$

and the unchanged fast equation (3.5) defined in both cases for $(E,n)\in {\mathbb {R}}^2$. Since all the results of Fenichel’s Theory are local around the subset of the critical manifold, we are focusing on, these extensions do not change the results.

Away from the fold point $p_F$ we determined above that the critical manifold is normally hyperbolic so, taking any compact subset of the left branch, we are able to apply Fenichel’s first Theorem 10 to perturb the attracting and repelling branches separately to locally invariant manifolds of the full system. Furthermore, by Fenichel’s second and third Theorems 11 and 12 the switching between the fast and the slow flow is also preserved for $\varepsilon >0$. Returning now to our original system (3.1) we can extend the fast fibres over $E=1$ using the continuity of the flow. Last it remains to prove that the switching at $p_F$ is preserved as well. We know that this point is a generic fold so we can do a coordinate transformation to normal form. Krupa and Szymolyan (2001a) presented in detail in the analysis of the normal form of a generic fold by performing a geometric blowup, a desingularisation technique first introduced by Dumortier (1978, 1993). Applying this method to our model concludes the proof. $\square$

##### Theorem 5

The heteroclinic segment of the critical manifold in the Karma model (3.1) with $I=0$ can be perturbed to a heteroclinic orbit between the equilibria for $\varepsilon >0$.

##### Proof (Sketch of the Proof)

Following the proof of Theorem 4, we are able to perturb any compact subset of the heteroclinic segment without the point at $E=1$ but we cannot directly guarantee that the left and right subsets connect. To demonstrate the existence of the expected heteroclinic orbit connecting the unstable equilibrium to the saddle, we need to look directly at the system with $\varepsilon >0$.

**Fig. 4:**
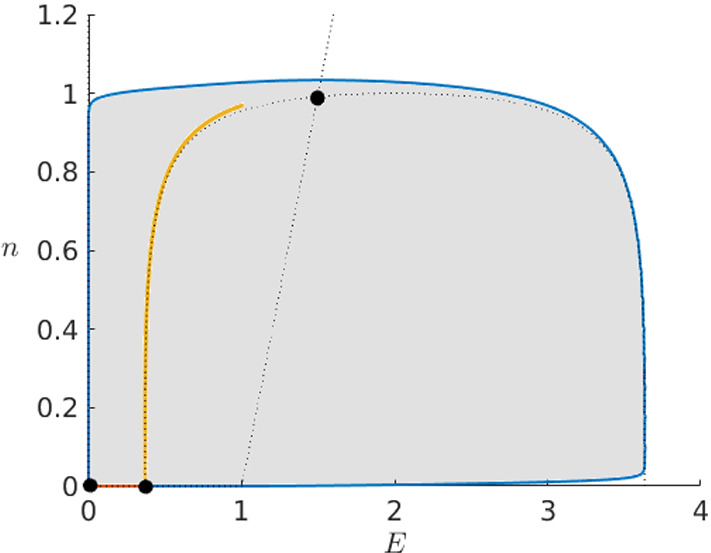
Plane (*E*, *n*) in the Karma model (3.1) for $\varepsilon >0$. In grey, we have the invariant set introduced in the proof of Theorem 5 enclosed by the unstable manifolds of the saddle (blue and orange). Furthermore, we have its stable manifold for $E<1$ (yellow), the *E*- and *n*-nullclines (dotted) and the equilibria of the system (black) (Colour figure online)

Since both unstable manifolds of the saddle converge by the analysis above to the origin, we are able to define an invariant set delimited by them as shown in Fig. 4. Furthermore, from the previous analysis we know that every orbit starting away from the heteroclinic segment will eventually converge to the origin so we follow that there is no periodic orbit and therefore no limit cycle in this set. Now we are able to apply the Poincaré–Bendixson Theorem in the limit $t\rightarrow -\infty$. Since the origin is unstable in backward time and there are no limit cycles, the theorem shows that in fact the now unstable manifold of the saddle needs to converge to the now stable equilibrium proving the existence of the expected heteroclinic orbit for positive $\varepsilon$. $\square$

##### Remark 2

To adapt Theorems 3–5 to the model with $\theta$ as introduced in (2.12) it is sufficient in Theorem 4 to divide the ${\mathbb {R}}^2$-plane into 3 regions instead of 2 separated by $E = 1$ and $E = a$ and perform the analysis above accordingly.

In summary, we have shown that although the general behaviour of the Karma ODE model is similar to the FHN ODE model, there are subtle mathematical differences, particularly in the case of using the standard variants in the literature.

#### Karma: External Current $I>0$

**Fig. 5:**
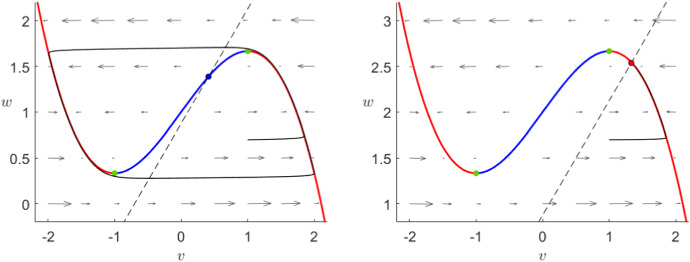
Phase plane of the FitzHugh–Nagumo system (3.2) for $\varepsilon =10^{-2}$ and $I=1$ (left) or $I=2$ (right). In both cases, we can see the critical manifold (3.3) divided by the fold points (green) into two attracting branches (red) and a repelling branch (blue) as well as the *w*-nullcline (dashed) and the unique fixed point, unstable for $I=1$ (black–blue) and stable for $I=2$ (black–red). Furthermore, we show a prototypical orbit (black) oscillating when $I=1$ and converging to the global equilibrium when $I=2$ (Colour figure online)

Next, we focus on the case where the external current $I>0$. In the FitzHugh–Nagumo model, it is well known that an external current shifts the critical manifold upwards as shown in Fig. 5. Without changing anything else, the dynamics switch from a stable resting state to an oscillatory behaviour to a stable depolarised state as the input *I* increases. To mathematically show these different behaviours, we can perform an analogous, yet more complicated, analysis as presented in Sect. 3.1.1 (Krupa and Szymolyan 2001b).

**Fig. 6:**
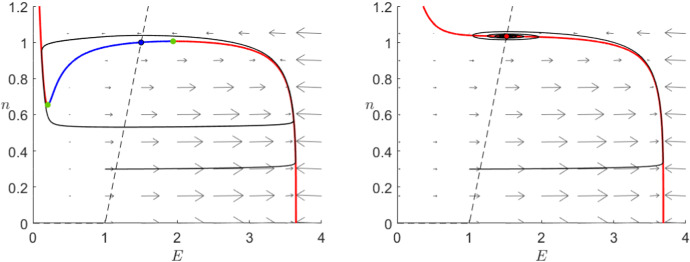
Phase plane of the Karma system (3.1) for $M=4$, $n_B=0.5$ $\varepsilon =10^{-2}$ and $I=0.1$ (left) or $I=0.5$ (right). In the case where $I=0.1$, we can see the critical manifold (3.9) divided by two fold points (green) into two attracting branches (red) and a repelling branch (blue) as well as the *w*-nullcline (dashed) and the unique unstable fixed point (black–blue). Furthermore, we show a prototypical orbit (black) oscillating. In the case where $I=0.5$, the unstable branch as well as the fold points have disappeared such that $C_0$ (red) is attracting everywhere and a prototypical orbit (black) converges to the unique stable equilibrium (black–red) (Colour figure online)

In contrast to that, adding an external current to the Karma model results in a significant change in the shape of the critical manifold as shown in Fig. 6. While we have a regime of *I* where the critical manifold is “S”-shaped comparable to FHN giving rise to similar relaxation oscillations, when *I* is big, the manifold flattens out in such a way that the curve is monotonous. In particular, this means that the model does not allow any relaxation oscillations or pulses for a high input *I*. Furthermore, in the Karma model the stable resting state disappears when it collides with the saddle in a fold bifurcation whereas in FHN only the stability of the already unique equilibrium changes. Lastly, the change of stability of the unstable node is for the most part independent of the shape of $C_0$. This means that, depending on the model parameters, we can observe bistability as well as a relaxation pulse with a stable depolarised state similar to FHN in addition to the dynamics we have already described.

The next theorem formalises all the different dynamic regimes described above.

##### Theorem 6

In the singular limit $\varepsilon =0$ of the Karma equations (3.1) with $I>0$, we have the critical manifold

3.9 $$\begin{aligned} C_0=\left\{ (E,n): n=\root M \of {E^*-\frac{E-I}{2E^2(1-\delta E)}}\right\} . \end{aligned}$$

Every candidate orbit can be constructed as concatenation of fast segments converging to one of the outer branches of $C_0$ which are attracting and slow segments on the critical manifold but we need to differentiate multiple parameter regimes for *I* giving rise to different overall dynamics.

The first threshold is given by $I=I_2$ where the equilibrium with $n>0$ changes from unstable to stable as *I* increases. Furthermore, we have

- $I<I_0\approx 0.08718$ The equations have three equilibrium points with a stable node and a saddle on the *E*-axis. If $I<I_2$ the behaviour is equivalent to the case $I=0$. Otherwise, the system is bistable.
- $I_0<I<I_1=\frac{4}{9}$ The equations have a unique equilibrium point. If $I<I_2$ the equilibrium is unstable and the system has a stable relaxation oscillation. Otherwise, the equilibrium is globally stable although there are relaxation pulses.
- $I>I_1(>I_2)$ The middle branch of $C_0$ disappears such that the critical manifold is attracting everywhere and the unique equilibrium is globally stable.

##### Proof (Sketch of the Proof)

Analogously to the previous section, we are now going to study separately the fast and slow subsystems in the singular limit in order to proof Theorem 6.

*Layer problem* The layer problem is defined by the equation

3.10 $$\begin{aligned} E'=-E+2(E^*-n^M)(E^2-\delta E^3)+I \end{aligned}$$

for *n* fixed. We can see directly that the derivative of the right-hand side is still given by (3.6). Since by definition any equilibrium is contained in $C_0$, we can plug in the equality

$$\begin{aligned} (E^*-n^M)=\frac{E-I}{2E^2(1-\delta E)} \end{aligned}$$

and rewrite that way the Jacobian depending on the external current *I* instead of *n* as follows

3.11 $$\begin{aligned} J(E;I)=-1+\frac{(E-I)(2-3\delta E)}{E(1-\delta E)}. \end{aligned}$$

To isolate any non-hyperbolic equilibrium of the fast system, we set $J(E;I)=0$ and obtain after simplifying

3.12 $$\begin{aligned} 0=2E^2-(\frac{1}{\delta }+3I)E+\frac{2}{\delta }I. \end{aligned}$$

Solving the quadratic equation for *E*, we find 2 curves of non-hyperbolic equilibria given by

3.13 $$\begin{aligned} E_\pm (I)=\frac{(4+3I)\pm \sqrt{(4+3I)^2-64I}}{4} \end{aligned}$$

which connect and disappear for $I\ge I_1:= \frac{4}{9}$. It is important to check for which values of *I* the curves $E_\pm (I)$ are in fact on the critical manifold, more precisely, whether

3.14 $$\begin{aligned} E^*-\frac{E_\pm -I}{2E_\pm ^2(1-\delta E_\pm )}\ge 0. \end{aligned}$$

The curve $E_+(I)$ satisfies this inequality for all $I\in [0,I_1]$ but for the curve $E_-(I)$ the inequality (3.14) is only satisfied when

$$\begin{aligned} I\in [I_0,I_1] \end{aligned}$$

with $$I_0 \approx 0.08718$$.

Having isolated the non-hyperbolic equilibria, we check that, similar to the previous section, when $I<I_1$ we have a division of the critical manifold into three separate branches where the Jacobian is negative on the outer ones and positive in the middle branch. When $I>I_1$, the Jacobian stays negative along the whole critical manifold.

*Reduced problem* The slow subsystem is still defined by (3.7) but now we have a different definition of $C_0$. The most important change lies in the fact that the *n*-nullcline will, due to continuity, cross the curve $E_\pm (I)$ in the (*E*, *n*)-plane for some $I_2\le I_1$ dependent on the system parameters $n_B$ and *M* as we increase *I*. By crossing this curve, the global equilibrium of the system (with $n>0$) changes its stability and becomes stable. Furthermore, we have already seen that the two equilibria at the *E*-axis collide and disappear for $I=I_0$ so that for $I>I_0$ we only have 1 equilibrium of the slow flow. $\square$

##### Remark 3

Looking at the full system, we identify $I=I_0$ as the bifurcation parameter where the system undergoes a saddle-node bifurcation when the 2 equilibria on the *E*-axis collide and disappear giving rise to the curve $E_-(I)$. For the corresponding values of *I*, we can check again that the conditions for a generic fold point are satisfied on both curves $E_\pm (I)$ everywhere except for the point $I=I_1$ and the singularity at $E_+(I_2)$ or $E_-(I_2)$. At the first one, the system undergoes a cusp bifurcation where the twofold points annihilate each other. We will come back to this bifurcation later on in more detail. Last, the intersection between the *n*-nullcline and $E_\pm (I)$ at $I=I_2$ satisfies the conditions of a nondegenerate fold but the slow flow is 0. We conclude that at this point we have a fold singularity.

Finally, similarly to the previous section, we construct the candidate orbits in the singular limit in the different parameter regimes.

- $I<I_0$ If $I<I_2$ the orbits are equivalent to without incoming current.
  If $I>I_2$, then the fast flow will converge to one of the attracting branches of $C_0$ but while every orbit on the left branch still converges to the origin, contrary to the previous case, all orbits on the right branch will stay on that branch converging to the second stable equilibrium. The slow flow on the repelling branch converges like before to the saddle point.
- $I_0<I<I_1$ First every orbit follows the fast fibres to one of the attracting branches of the critical manifold.
  If $I<I_2$, the slow flow leads then to the next fold point where we can again use a fast fibre to jump to the other attracting branch forming a cycle. The flow on the repelling branch will converge away from the equilibrium to the folds following from there the cycle.
  If $I>I_2$ and assuming the *n*-nullcline crosses $E_+(I)$, then the flow on the left and middle branch will still converge to the minimum jumping to the right branch. There all orbits converge to the equilibrium. The case where the *n*-nullcline intersects $E_-(I)$ is equivalent subject to interchange left and right and taking the maximum instead of minimum.
- $I_1<I$ The entire critical manifold is attracting so every orbit flows fast to it and then converges to the unique equilibrium.

##### Remark 4

To go briefly into the biophysical implications of the above observations, we note that all $I_0,I_1,I_2$ are important thresholds affecting differently the behaviour of the cell. Whenever we have a background stimulation $I>I_2$, any cell which depolarises over this threshold would not be able to repolarise anymore and will therefore cease to “fire” further signals. On the other hand, a background stimulation $I_0<I<I_1$ and $I<I_2$ results in a self-excitatory system which will “fire” regularly. Finally, when the background stimulation is higher than $I_1$ the cell will automatically depolarise so that any future signal is blocked.

##### Theorem 7

Whenever $E_-(I)\ne 1$, candidate orbits found in the singular limit $\varepsilon =0$ of equations (3.1) with $I>0$ away from the bifurcation points $I_0$ and $I_2$ can be perturbed to solution curves of the full system with $\varepsilon >0$.

##### Proof (Sketch of the Proof)

Analogously to Theorem 4, we find that also for $I>0$ away from the intersection between $E=1$ and the critical manifold we can perturb every orbit as expected for $\varepsilon >0$. In the case when $E_-(I)>1$, in particular when $I>I_1$, this point lies in the left branch of $C_0$. After continuing the slow manifold obtained for $E<1$ over this line, we can use the attracting properties of the slow manifold for $E>1$ to follow that both manifolds will approach each other. Recalling that the slow manifold is not unique we can directly choose the continuation of the left part to also be our representative slow manifold for $E\ge 1$. To finish the proof, we need to separate the different parameter regimes when $E_-(I)<1$. If we first take $I<I_2$, we have the following cases.

- When $I<I_0$, the system is equivalent to the case with $I=0$ and the proof of Theorem 5 can still be applied to derive the heteroclinic orbit between the unstable node and the saddle point.
- When $I_0>I>I_1$, we have already derived a stable limit cycle with the unstable fixed point the only orbit not converging to it. In particular, we know there are no further periodic orbits inside the limit cycle. This means that, defining an invariant set delimited by the cycle, we can use the Poincaré–Bendixson Theorem to show that the segment of repelling slow manifold with $E>1$ will converge to the limit cycle for $t\rightarrow \infty$ as well as that the segment with $E<1$ will converge to the equilibrium for $t\rightarrow -\infty$.

Finally, we look at the system with $I>I_2$. By reversing time, the repelling branch of the critical manifold becomes attracting and so we can use the same technique applied above and choose the continuation of the left segment of the middle branch as slow manifold. Following the analysis given by Fenichel’s theorems and geometric blowup, we follow that the middle branch of the slow manifold flows over the fold point diverging in backward time. In the case where $I<I_0$, this manifold defines a separatrix dividing the phase space into the basins of attraction of the two stable equilibria. In the case where $I>I_0$, this slow manifold separates the orbits converging directly to the stable equilibria and the orbits which perform a relaxation pulse over the left or right branch of $C_0$ and one of the fold points before converging. $\square$

The limit case with $E_-(I)=1$ cannot be analysed with the methods used above since the geometric blowup also requires higher regularity. By continuity, we would expect that we can still perturb the candidate orbits for $\varepsilon >0$ but this still has to be proven rigorously. Furthermore, when $I=I_0$ or $I=I_2$ the system has folded singularities. It is known that in small neighbourhoods around these points we can find canards and so-called canard explosions. For more details about these solutions, see Dumortier and Roussarie (1996), Krupa and Szymolyan (2001a, 2001b), Kuehn (2015).

##### Remark 5

Similar to the previous section, if we want to adapt the theorems above to the function (2.12) we only need to take into account the extra non-differentiable point. Note that, although the position of the equilibrium for $n > 0$ and therefore the bifurcation point $I_2$ depend additionally on the parameter *a*, this does not further affect the analysis. Looking closely we see that they do not depend directly on *a* but only on the product $a\cdot n_{B}$. Therefore, since *a* is assumed to be small, we have in fact already considered all the possible values for $I_2$ due to the dependence on the parameter $n_{B}$.

##### Remark 6

All the existence results obtained by Fenichel’s Theory require $\varepsilon$ to be “small enough”. In applications, we need to check for every case independently what “small enough” means specifically.

By looking at simulations, we see that when $I<I_2$ the orbits behave as expected even for relatively large $\varepsilon \approx 10^{-1}$. Nevertheless, when the equilibrium changes stability for $I=I_2$, the orbits oscillate around the equilibrium instead of converging through a slow manifold as expected from Fenichel’s Theory even for very small $\varepsilon \approx 10^{-4}$. This shows that even knowing that there exists an $\varepsilon$ for which this theory is applicable, for some values it is not the case. To understand what actually happens at this point with reasonable $\varepsilon$, we have to look at the eigenvalues of the fast subsystem as well as of the full system.

Although the Jacobian *J* of the fast subsystem is strictly smaller than 0, the critical manifold stays very close to non-hyperbolicity and so the absolute value of *J* is very small. If we calculate the eigenvalues of the full system at the unique equilibrium, we have:

$$\begin{aligned} \lambda _\pm = \frac{J-\varepsilon }{2} \pm \frac{1}{2}\sqrt{(J-\varepsilon )^2-4\varepsilon \left( -J+\frac{2}{n_B}Mn^{M-1}(E-1)(E^2-\frac{1}{4}E^3)\right) }. \end{aligned}$$

It holds that $J<0$ and the equilibrium is away from $E=1$ and $E=4$ therefore we see that the parenthesis in the second term of the discriminant is always positive and of order 1 w.r.t. $\varepsilon$ so the whole summand is in ${\mathscr {O}}(\varepsilon )$. Nevertheless, if $J\in {\mathscr {O}}(\varepsilon )$ then the first summand is of order $\varepsilon ^2$ such that the eigenvalues become complex and our equilibrium is a stable spiral instead of a stable node.

Nevertheless, the equilibrium is still globally stable and every orbit will eventually converge to it.

#### Extended System (*E*, *n*, *I*)

We have shown above that both models exhibit relatively similar qualitative behaviour when considering *I* a fixed parameter. Now, we shortly investigate an extended 3-dimensional systems with the additional equation

**Fig. 7:**
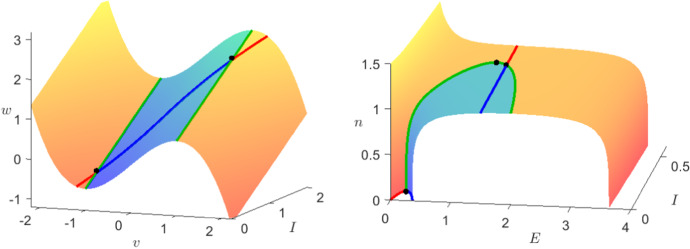
Critical manifold (3.3) for the extended FitzHugh–Nagumo model (3.2) in the (*v*, *w*, *I*)-space (left) and (3.9) for the extended Karma model (3.1) with $M=4$, $n_B=0.5$ and $\varepsilon =10^{-2}$ in the (*E*, *n*, *I*)-space (right), in both cases with the additional slow equation (3.15) setting $f=0$. The critical manifolds are divided by the fold curves (green) into attracting (orange) and repelling (blue) regions. Furthermore, we see the curves of stable (red) and unstable (blue) equilibria as well as the bifurcation points (black) (Colour figure online)

3.15 $$\begin{aligned} I'=f(v,w,\varepsilon )\quad \text { or }\quad I'=f(E,n,\varepsilon ) \end{aligned}$$

for some sufficiently smooth function $f:{\mathbb {R}}^3\rightarrow {\mathbb {R}}$. By choosing $f=0$, we can find our models (3.2) and (3.1) as special cases. Figure 7 shows the critical manifolds for the Karma model as well as FitzHugh–Nagumo in this extended setting.

This representation allows for proper analysis of the fold curves. Although both models show exactly two curves of folds, their behaviour is clearly very different. Firstly, we notice the extreme sensitivity of the Karma model to external currents close to $I_0$ which is not present for FHN. Furthermore, both fold curves in the FitzHugh–Nagumo model are parallel to each other while in the Karma model they collide and disappear like we had seen above. This collision point $I=I_1$, $E=\frac{4}{3}$ and the corresponding *n* given by (3.9) defines a cusp bifurcation in the Karma model. It is clearly a non-hyperbolic point and therefore it cannot be analysed using classical Fenichel theory. Nevertheless, we can do a similar analysis as for a fold point using a coordinate transformation to normal form and geometric blowup. A detailed analysis of a cusp point using these techniques was presented by Broer et al. (2013).

In general, the existence of a cusp singularity presents the possibility for a diverse set of behaviours if we consider a slowly changing external current, e.g. a slow periodic input. By extending the Karma model considering a change in *I*, we get the possibility of relaxation oscillations with a smooth return. This means that we can have oscillations whereby after a fast jump we are able to return to our starting point following only the slow dynamics. This type of behaviour is not possible in the FitzHugh–Nagumo model even after allowing changes in *I*.

##### Remark 7

The analysis presented in Broer et al. (2013) assumes that the bifurcation point is not an equilibrium of the full system. In the Karma model, this is in general the case, nevertheless we have the case where $I_1=I_2$ when the cusp point is in fact a global equilibrium. This case has (to our knowledge) not been analysed mathematically yet and would be an interesting future extension to the current analysis.

### Travelling Waves

As the next step in our analysis, we want to consider also the diffusion in the models concentrating on the existence of a travelling pulse in the 1D case. Like before, the existence as well as stability of travelling waves for the FitzHugh–Nagumo equations has been studied extensively (Flores 1991; Hastings 1976; Jones 1984). We are particularly interested in the construction of pulse solutions performed by Guckenheimer and Kuehn (2009). The authors looked at the asymmetric FHN equations

3.16 $$\begin{aligned} \begin{aligned} \frac{\partial v}{\partial t}&= D\varDelta v + v(v-a)(1-v)-w+I\\ \frac{\partial w}{\partial t}&= \varepsilon (v-\gamma w) \end{aligned} \end{aligned}$$

with the parameter values $\gamma =1$, $a=\frac{1}{10}$ and $D=5$. The system is very similarly to (1.1) also controlled by a cubic critical manifold in the ODE case. When we add the diffusion term, the system exhibits travelling pulse solutions which they proved using a numerical continuation method for the fast fibres in the co-moving frame. In the parameter space (*w*, *c*), the authors found a “V”-shaped curve of fast heteroclinic fibres connecting the left and right branches of the critical manifold. When $c=0$, the system is Hamiltonian and there is a $w=w_*$ such that there is a double heteroclinic orbit. When *w* is smaller than $w_*$ we have a connection from the left branch to the right one while when *w* is bigger the connection goes in the opposite direction. A concatenation of this fibres combined with the slow flow on the critical manifold can then be perturbed analogously to the previous section for $\varepsilon >0$, although the technical details become mathematically very involved.

Here, we carry out a similar analysis for the Karma model (2.13) starting by introducing the corresponding co-moving frame $z=x+ct$ such that the equations are now given by

3.17 $$\begin{aligned} \begin{aligned} cE_z&=DE_{zz}-E+2\left( E^*-n^M\right) (E^2-\delta E^3)+I\\ cn_z&= \varepsilon \left( \frac{1}{n_B}\theta (E-1)-n\right) . \end{aligned} \end{aligned}$$

We can easily transform the model into a first-order system by introducing an additional variable *w*

3.18 $$\begin{aligned} \begin{aligned} E_z&= w\\ Dw_z&=cw+E-2\left( E^*-n^M\right) (E^2-\delta E^3)-I\\ cn_z&=\varepsilon \left( \frac{1}{n_B}\theta (E-1)-n\right) . \end{aligned} \end{aligned}$$

We now have two additional parameters with respect to the ODE model, namely *c* and *D*. The parameter *c* gives the velocity at which the travelling wave moves. Changing the sign of the parameter *c* is equivalent to inverting the direction of the wave variable *z* and substituting *w* by $-w$. Therefore, without loss of generality we can restrict our analysis to $c>0$.

The second parameter *D* is the diffusion coefficient. For this parameter, there are different scalings often used in the literature. Specifically in the original papers introducing the Karma model, the author presents a diffusion coefficient $D\in {\mathscr {O}}(\varepsilon )$, introducing therefore a third scale to the system (Karma 1993) while a constant diffusion $D\in {\mathscr {O}}(1)$ was used in Karma (1994).

Below we focus on the model with $D\in {\mathscr {O}}(1)$ and for simplicity only the case without incoming current $I=0$. In the following theorems, we want to illustrate that the Karma model (2.13) can exhibit a travelling pulse solution with the resting state (0, 0) as start and end state.

#### Theorem 8

In the singular limit $\varepsilon =0$, there exists a homoclinic candidate orbit to equations (3.17) satisfying the asymptotic conditions

3.19 $$\begin{aligned} \lim _{z\rightarrow \pm \infty }(E(z),n(z))=(0,0). \end{aligned}$$

We sketch the geometric idea of the proof of this result. The model, after transformation to the first-order system (3.18), is a (2, 1)-fast–slow system with one-dimensional critical manifold given by

3.20 $$\begin{aligned} C_0=\left\{ (E,w,n): w=0,~E=0 \text { or } n=\root M \of {E^*-\frac{1}{2E(1-\delta E)}}\right\} . \end{aligned}$$

*Reduced system* The slow flow on $C_0$ differs from the one in the ODE model only by a factor $\frac{1}{c}$ so we are simply scaling the flow. In particular, we have the same global equilibria as before embedded into the (*E*, *n*)-plane.

*Layer problem* The fast subsystem is defined by the equations

3.21 $$\begin{aligned} \begin{aligned} E'&= w\\ Dw'&= cw+E-2\left( E^*-n^M\right) (E^2-\delta E^3). \end{aligned} \end{aligned}$$

The equilibria correspond to the points on the critical manifold for the different values of *n*. By choosing a different representation, we have the fixed point $p_0=(0,0)$ and for $n^M\le 1.0415$

$$\begin{aligned} p_1=\left( 2-2\sqrt{1-\frac{1}{2(E^*-n^M)}},0\right) , \quad p_2=\left( 2+2\sqrt{1-\frac{1}{2(E^*-n^M)}},0\right) . \end{aligned}$$

The Jacobian at this points is given by

3.22 $$\begin{aligned} J(E,w)=\begin{pmatrix} 0&{}1\\ \frac{1}{D}[1-2(E^*-n^M)(2E-3\delta E^2)]&{}\frac{c}{D} \end{pmatrix} \end{aligned}$$

with eigenvalues

$$\begin{aligned} \lambda _\pm =\frac{c}{2D}\pm \sqrt{\frac{c^2}{4D^2}+\frac{1}{D}[1-2(E^*-n^M)(2E-3\delta E^2)]} \end{aligned}$$

and, when $\lambda _\pm$ are real, corresponding eigenvectors

$$\begin{aligned} v_\pm =\begin{pmatrix}1\\ \lambda _\pm \end{pmatrix}. \end{aligned}$$

We can directly check that the equilibria $p_0$ and $p_2$ are saddles and $p_1$ is unstable. In addition, we know that $p_1$ is a node when

$$\begin{aligned} c^2> 4D\left[ 2-4(E^*-n^M)\left( 1-\sqrt{1-\frac{1}{E^*-n^M}}\right) \right] \end{aligned}$$

and a spiral otherwise.

Given the local structure around the critical manifold, we want to find heteroclinic connections between $p_0$ and $p_2$ to later combine with the slow flow to heteroclinic candidate orbits.

#### Lemma 1

For equation (3.21), it holds that

(i) For every $n\in [0,1]$, there exists a $c>0$ such that the system has a heteroclinic connection. When $n<\root M \of {15/16}$, the orbit flows from $p_0$ to $p_2$ while for $n>\root M \of {15/16}$ the orbit flows from $p_2$ to $p_0$. At $n=\root M \of {15/16}$, the system has a double heteroclinic orbit in the limit $c=0$.
(ii) For $n=1$, there exists a $c_{min}$ such that for every $c\ge c_{min}$ the system has a heteroclinic connection from $p_2$ to $p_0$.

#### Proof (Sketch of proof of (i))

In order to prove the first statement, we are going to follow the strategy in Guckenheimer and Kuehn (2009). Our first step is to compute the stable and unstable manifolds of $p_0$ and $p_2$ by taking initial conditions close to the equilibria on their tangent spaces. Next, we define the plane $\varSigma$ where $E=\frac{E_2}{2}$ and calculate the intersection points $q_0$ and $q_2$ with the previously computed orbits depending on $n^M$ and *c*. The zeros of the function

**Fig. 8:**
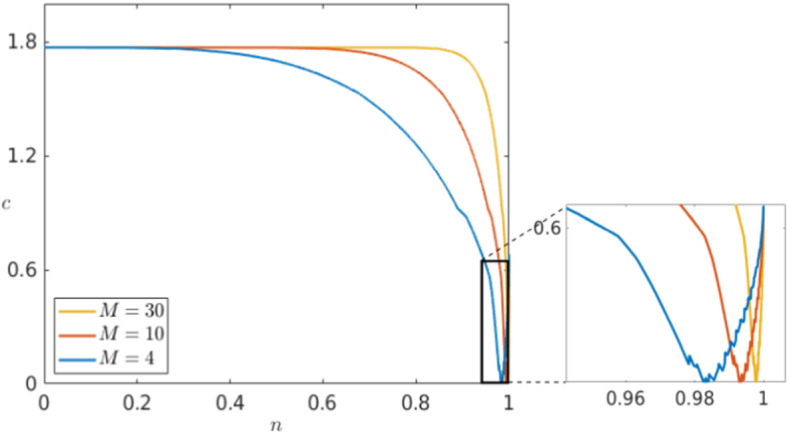
Heteroclinic orbits of the fast subsystem (3.21) of the Karma model in the co-moving frame for $D=1$ and different values of *M* in the parameter space (*n*, *c*). The point $(n,c)=(\root M \of {15/16},0)$ divides the curve of heteroclinic orbits into two separate parameter regimes and therefore also branches. The left branch with $n<\root M \of {15/16}$ corresponds to orbits connecting the origin to $p_2$ as $z\rightarrow \infty$ while on the right branch with $n>\root M \of {15/16}$ we have heteroclinic orbits connecting the equilibria in the opposite direction (see close-up) (Colour figure online)

3.23 $$\begin{aligned} \varDelta (n^M,c)=q_0(n^M,c)-q_2(n^M,c) \end{aligned}$$

define finally the parameters which give rise to heteroclinic orbits in the fast subsystem. Once we have one such parameter pair, the complete curve in the parameter space can be found because of continuity by slowly changing $n^M$ and computing again the zeros of $\varDelta$. Figure 8 shows the computed zeros.

The left branch of zeros reaching from $n=0$ to $n=\root M \of {15/16}$ corresponds to the intersection of the unstable manifold of $p_0$ with the stable manifold of $p_2$. The right branch (see close-up) corresponds to the unstable manifold of $p_2$ intersecting the stable manifold of $p_0$. This numerical computation could then be made rigorous, e.g. via employing rigorous numerical techniques, which are already well established in the context of FHN (Arioli and Koch 2015), which concludes the proof of first part of statement (i). For the last part of the statement, we observe that in the limit $c=0$ the fast subsystem is Hamiltonian with the first integral given by

3.24 $$\begin{aligned} H(E,w)=\frac{1}{2}w^2-\frac{1}{2D}E^2+\frac{2}{D}(E^*-n^M)\left( \frac{1}{3}E^3-\frac{\delta }{4}E^4\right) . \end{aligned}$$

**Fig. 9:**
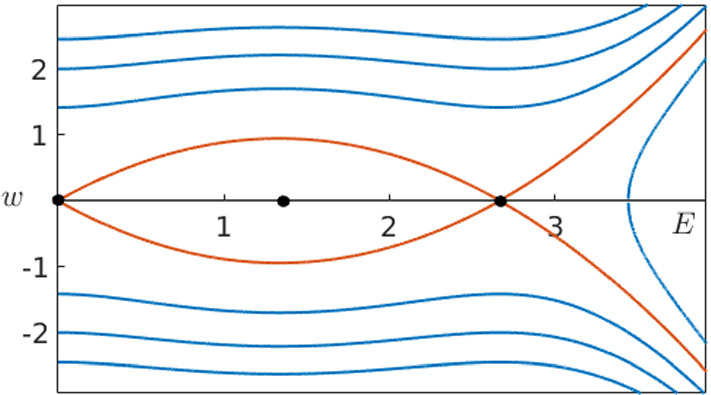
Energy levels (3.24) (blue) of the fast subsystem (3.21) of the Karma model in the co-moving frame for $D=1$ when $c=0$ and $n^M=15/16$ showing a double heteroclinic orbit connecting $p_0=(0,0)$ and $p_2$ at $H(E,w)=0$ (orange) (Colour figure online)

We calculate directly that the energy level at the origin is always 0 and $H(p_2)=0$ holds if and only if $n^M=\frac{15}{16}$. Together with the results illustrated in Fig. 8, this strongly indicates that for $(n^M,c)=(15/16,0)$ the system has a double heteroclinic orbit. We can confirm this by computing the energy level $H(E,w)=0$ as shown in Fig. 9.$\square$

Before we continue illustrating the geometric ideas behind the proof of the second part of Lemma 1, we want to make a remark regarding the previous construction.

#### Remark 8

In Fig. 8, we can see the curve of heteroclinic orbits for different values of *M*. In particular, we can see the insensitivity of the wave-front velocity with respect to the slow variable *n* when $M\gg 1$ which is one of the important advantages mentioned in Karma (1993) of the Noble and Karma model over FitzHugh–Nagumo.

#### Remark 9

Deng (1991) proved that in the FitzHugh–Nagumo model, under certain conditions, the perturbation of a double heteroclinic orbit in the full system can result in infinitely many front and back wave solutions with an arbitrary number of oscillations. Although his results are not directly applicable in our situation as we would have to adjust the slow variable nullcline to obtain two full system equilibria on the two saddle-type branches, the existence of a double fast subsystem heteroclinic orbit in the Karma model clearly indicates already the possibility of more complex travelling waves than just single pulses.

#### Proof (Sketch of proof of (ii))

**Fig. 10:**
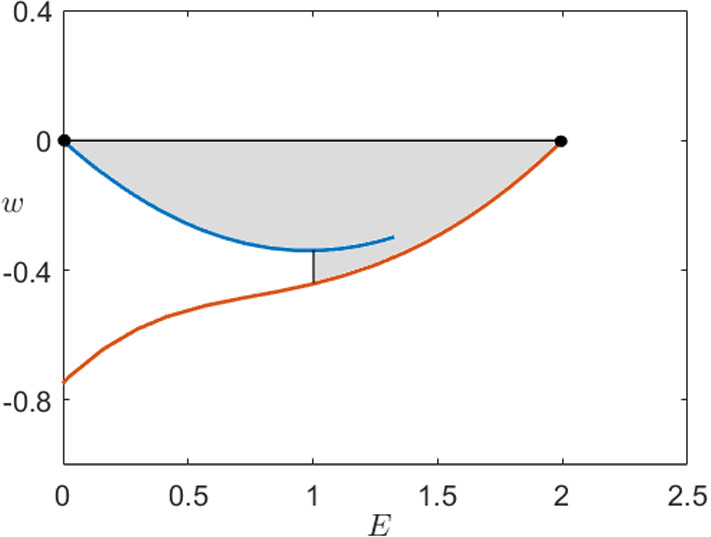
Phase plane (*E*, *w*) of the fast subsystem (3.21) of the Karma model in the co-moving frame for $D=1$ when $n=1$ and $c>c_{min}$. We can observe the unstable manifold of $p_0$ (blue) as well as the stable manifold of $p_2$ (orange). Furthermore, we see the negatively invariant set (grey) introduced in the proof of Lemma 1 (ii) enclosed by the invariant manifolds, the *E*-axis and the line segment between them at $E=1$ (Colour figure online)

We have seen in the previous part that the unstable manifold of $p_2$ and the stable manifold of $p_0$ connect uniquely for $c=c_{min}\approx 0.707$. By continuity, for every $c>c_{min}$ we find a negatively invariant set enclosed by the *E*-axis, the stable manifold of $p_0$ and unstable manifold of $p_2$ and the vertical segment connecting them at $E=1$ as shown in Fig. 10. Since we know there are no further equilibria in this set and therefore also no limit cycle, we can apply the Poincaré–Bendixson Theorem to obtain that the stable manifold of $p_0$ converges for $t\rightarrow -\infty$ to $p_2$ through the centre manifold giving rise to further heteroclinic connections from $p_2$ to $p_0$. $\square$

#### Proof (Sketch of Proof of Theorem 8 (continued))

We can now easily construct a singular candidate orbit combining the slow and fast segments. Starting at the origin as the resting state, we can jump to $p_2$ by a fast fibre where we follow the slow flow upwards. Since we jumped with $c\approx 1.77$, we cannot jump until we reach the fold point at $n=1$. Using the additional fast fibres we identified above, we are able to jump back to $p_0$ and there follow the slow flow towards the origin. $\square$

#### Theorem 9

The homoclinic candidate orbit found in the singular limit $\varepsilon =0$ of equations (3.17) can be perturbed to a homoclinic solution of the full system with $\varepsilon >0$.

#### Proof (Idea of proof)

The transition from the singular limit to the regular case can be done analogously to Sect. 3.1. Away from $E=1$ where the system is not smooth and the non-hyperbolic fold point (2, 0, 1), we can apply Fenichel’s Theory (Theorems 10–12) to obtain the corresponding orbit in the regular case. Again, we can extend the orbits for $E=1$ by continuity since we know that we are away from the critical manifold and finally the fold point can be analysed using geometric blowup (Dumortier 1978, 1993; Krupa and Szymolyan 2001a). $\square$

#### Remark 10

Since the fast subsystem in the model with $\theta$ as in (2.12) is the same, the existence of the heteroclinic orbits, including their specific speed *c*, still holds. Therefore, the only difference in regard to the proof of the existence and specific construction of travelling waves between both choices of $\theta$ is that we need to construct the orbit using continuity instead of Fenichel’s Theorems at the second non-differentiable point $E = a$.

We recall that in the FitzHugh–Nagumo model a travelling wave will jump to a fast fibre directly from the normally hyperbolic part of the critical manifold. We have now shown that in contrast to that a pulse solution for Karma model needs the jump segments generated by the fold point through the centre manifold. This is a key difference between the two models. It results in a fixed position of the wave back in the phase space and a slower repolarisation than depolarisation rate which Karma already identified as important properties for cardiomyocytes (Karma 1993).

## Numerical Simulations

**Fig. 11:**
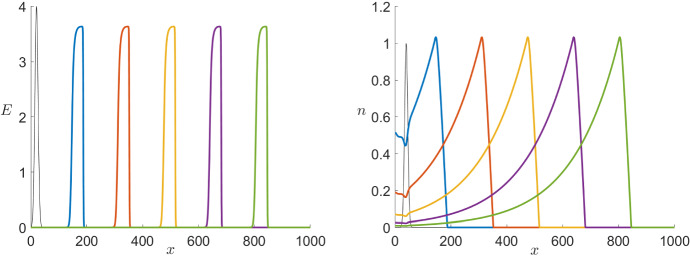
Simulation of the Karma PDE model (2.13) showing the time steps $t=100,200,300,400$ and 500 for both variables *E* (left) and *n* (right). In both cases, the initial conditions (black) where chosen as bump functions centred at $x=50$ and we see pulse solutions propagating to the right (Colour figure online)

In this section, we simulate the full PDE systems with a focus on the Karma model. In particular, we want to interpret the numerical simulations in relation to the analysis presented above in order to understand the PDE dynamics (Kuehn 2019) we can actually observe. For this, we will use the parameter values $\varepsilon =10^{-2}$, $D=1$, $M=4$, $n_B=0.5$ and $I=0$ except explicitly mentioned otherwise. Figure 11 shows the evolution of the system initialised with a bump function centred at $x=50$.

For the Karma model, we see from Fig. 11 that in fact for a big enough region in *x* the dynamics converge to a travelling pulse as we have found analytically. Since the simulations converge to a travelling wave given an arbitrary initial profile it (most likely) follows that the travelling pulse is at least locally asymptotically stable and that it does have a substantial basin of attraction. We have not proven the local asymptotic stability analytically here but this would be an interesting point in future work as it is well known that the FHN PDE has wide parameter ranges, where stable pulses occur and where geometric techniques allow us to prove stability (Jones 1984; Jones et al. 1991).

**Fig. 12:**
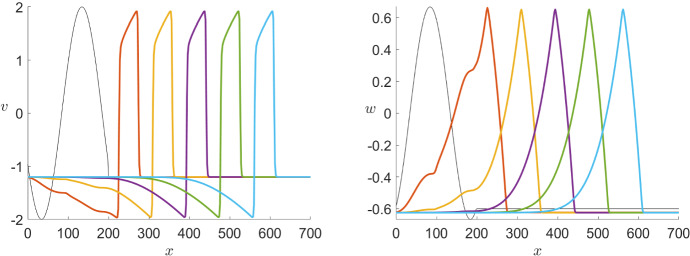
Simulation of the FitzHugh–Nagumo PDE model (1.1) showing the time steps $t=100,200,300,400$ and 500 for both variables *v* (left) and *w* (right). In both cases, the initial conditions (black) where chosen as pulses with small overshoots and we see pulse solutions propagating to the right where *v* maintains the overshoot while the wave for *w* does not (Colour figure online)

As a comparison, Fig. 12 shows a similar simulation for the FitzHugh–Nagumo model (1.1) with $\varepsilon =10^{-2}$, $D=1$ and $I=0$.

**Fig. 13:**
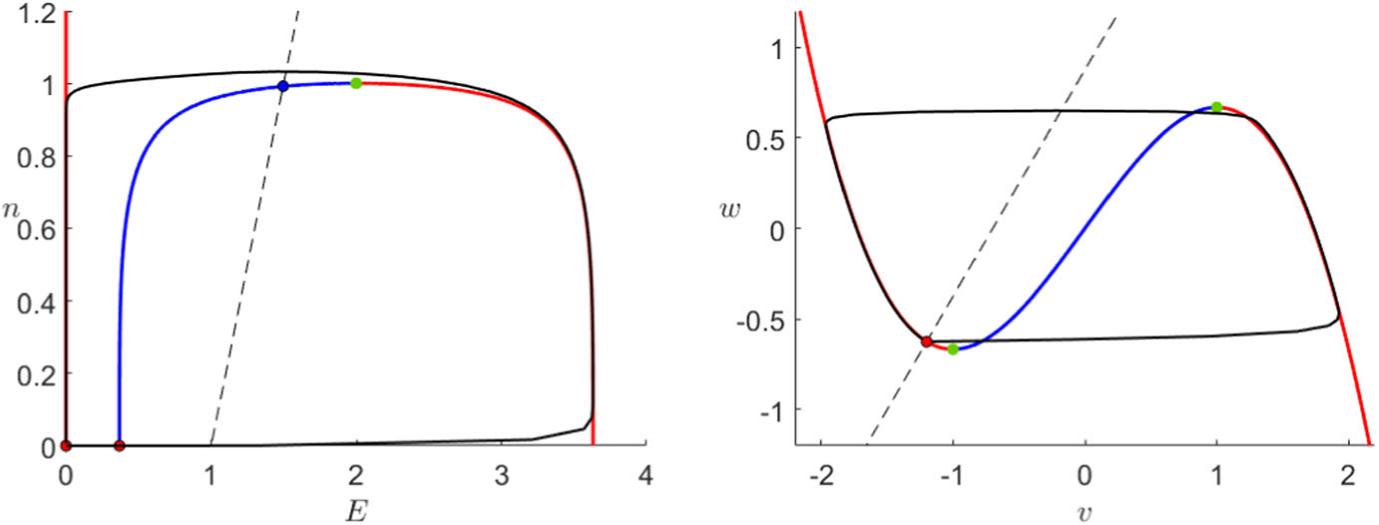
Projection of the pulse solutions (black) of the PDE models of Karma (2.13) (left) and FitzHugh–Nagumo (1.1) (right) at $t=500$ onto the (*E*, *n*)-plane or the (*v*, *w*)-plane, respectively. Furthermore, we have the critical manifold divided into branches of saddle type (red) and unstable branches (blue) as well as the fold points (green) and the saddles (black–red) and unstable equilibria (black–blue). We see that while FHN jumps between the branches of saddle type away from the folds the pulse for Karma runs over the fold point (Colour figure online)

At first sight, we see that a big difference between Karma and FitzHugh–Nagumo is the hyperpolarisation present only in the second model. Although there are heart tissues which show hyperpolarisation, if we want to model, e.g. ventricular cells the representation in the Karma model is notably more accurate. Furthermore, we recall that the repolarisation jump of the travelling wave we constructed in the previous section is ignited differently in both models, once on the fold point and once on the hyperbolic part of the manifold. Figure 13 shows that this is the case as well for the limit wave in the full PDE model. As stated before, this is the reason for the slower recovery rate in the Karma equations which gives us a key difference between both models.

**Fig. 14:**
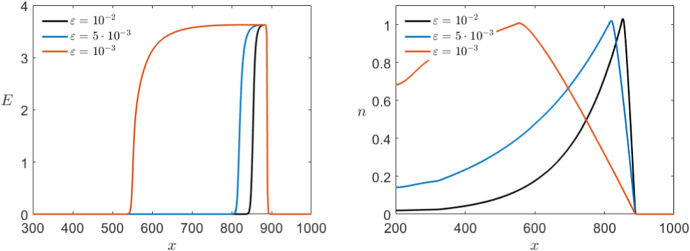
Comparison of the effect of multiple values of $\varepsilon$ in a simulation of the Karma equations (2.13) with the same bump initial conditions shown in Fig. 11 for both variables *E* (left) and *n* (right) via a time shot at $t=500$ (Colour figure online)

To finish the numerical analysis, we want to take a closer look at the effect of other parameters involved in the models and look first at $\varepsilon$. We start with the Karma model. Following the values introduced in Karma (1993, 1994) we have chosen for our simulations $\varepsilon =10^{-2}$ as our basis value. In addition, to make sure that the analysis above holds and we have in fact a travelling pulse solution, we need $\varepsilon$ to be small enough. Increasing $\varepsilon$ shows that already for $\varepsilon =0.08$ the travelling pulse dynamics seems to break down. Therefore, we will focus on smaller values of $\varepsilon$. By simulating the model with lower values, we notice that, as expected, *n* becomes slower as we decrease $\varepsilon$ so that the pulses for *E* as well as *n* elongate (see Fig. 14). Further, we observe in the right panel that the convergence speed towards the travelling pulse is much slower for smaller $\varepsilon$. Nevertheless, the wave speed appears to stay unchanged for different values of $\varepsilon$. Since we analytically demonstrated a geometric construction for the existence of the travelling pulses taking the wave speed *c* as a parameter we would in fact expect changes in *c* of order $\varepsilon$ with *c* converging to the constant value $\approx 1.77$ as $\varepsilon \rightarrow 0$. It is also intuitively clear from a biological point of view that the wave speed should depend on the properties of the medium, e.g. the diffusion *D*, but be quite independent of the cells recovery speed.

**Fig. 15:**
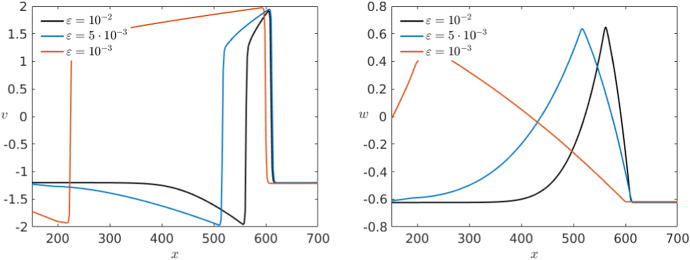
Comparison of the effect of multiple values of $\varepsilon$ in a simulation of the FitzHugh–Nagumo equations (1.1) with the same initial conditions shown in Fig. 12 for both variables *v* (left) and *w* (right) via a time shot at $t=500$ (Colour figure online)

Again we can compare this with the effects of varying $\varepsilon$ in the FitzHugh–Nagumo model shown in Fig. 15. Overall, the effect of varying $\varepsilon$ observed in both models is similar. Nevertheless, for $\varepsilon =10^{-3}$ we find a change in the wave speed in the FitzHugh–Nagumo model while, as mentioned above, is not visible for the Karma model.

**Fig. 16:**
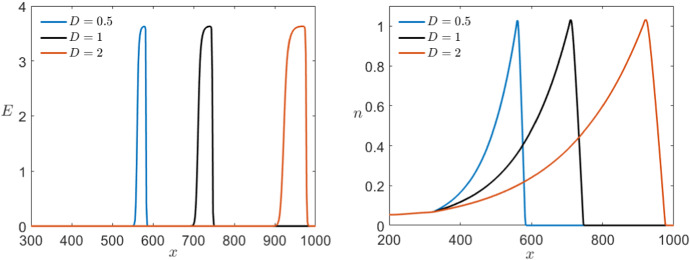
Comparison of the effect of multiple values of *D* in a simulation of the Karma equations (2.13) with the same bump initial conditions shown in Fig. 11 for both variables *E* (left) and *n* (right) via a time shot at $t=400$ (Colour figure online)

We now want to consider the effects of different diffusion coefficients *D* again starting with the Karma model. As mentioned before, we use as basis value for the diffusion $D=1$ for simplicity although the value used in Karma (1994) is 2.75. In particular, we would like to make sure that $D\in {\mathscr {O}}(1)$ so that the previous analysis applies. Specifically for our model with $\varepsilon =10^{-2}$, our simulations lead to assume that $D>0.11$ since otherwise the pulse seems to disappear. In Fig. 16, we consider three different simulations starting with the same initial conditions for different diffusion coefficients in the range of interest. We see that in this case the wave velocity is as expected strongly affected. An increase in the diffusion rate leads to higher wave velocity. Furthermore, we also see that a bigger diffusion coefficient also results in a slightly longer pulse.

**Fig. 17:**
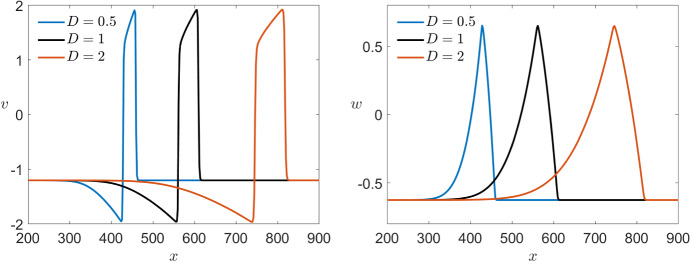
Comparison of the effect of multiple values of *D* in a simulation of the FitzHugh–Nagumo equations (1.1) with the same initial conditions shown in Fig. 12 for both variables *v* (left) and *w* (right) via a time shot at $t=450$ (Colour figure online)

In the corresponding simulation of the FitzHugh–Nagumo in Fig. 17, we see that the effects of different diffusion coefficients on both models are equivalent.

**Fig. 18:**
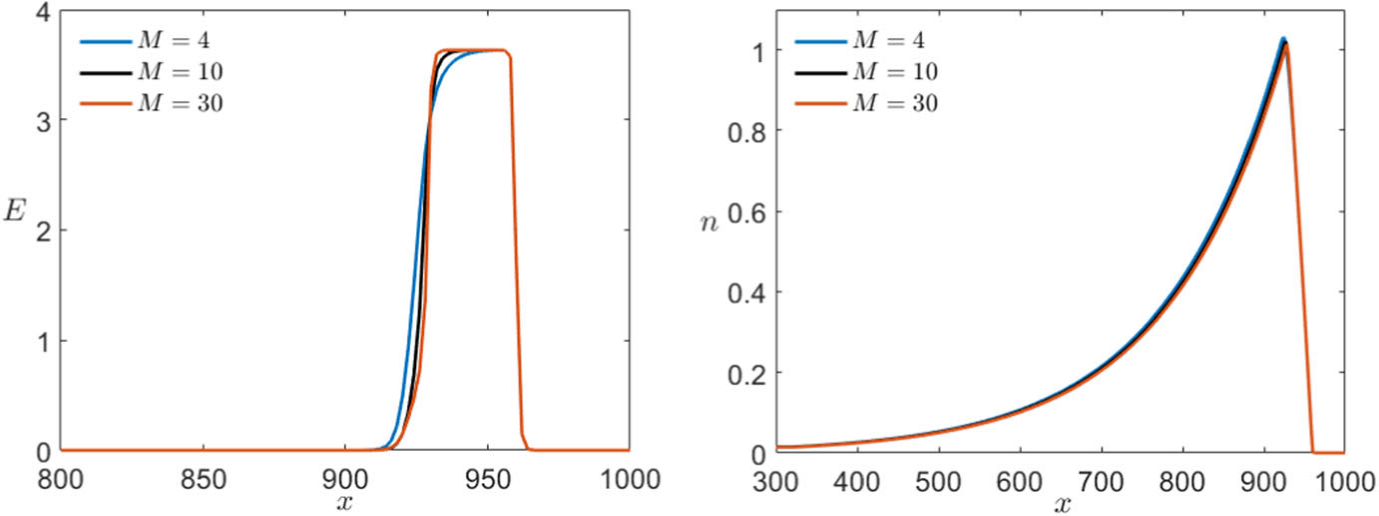
Comparison of the effect of multiple values of *M* in a simulation of the Karma equations (2.13) with the same bump initial conditions shown in Fig. 11 for both variables *E* (left) and *n* (right) via a time shot at $t=550$ (Colour figure online)

Similarly, we can look at the control parameters *M* and $n_B$ specific to Karma which are not fixed a priori. Using as a starting point again the values introduced by Karma (1993, 1994), we follow the range of interest for the parameter *M* that from modelling point of view varies from $M=4$ up to $M=30$. Even so, a higher or lower value does not qualitatively change the dynamics of the system. In Fig. 18, we see that *M* has almost no effect on the dynamics of the slow variable *n* but controls the sharpness of the pulse for *E*. From biophysical modelling point of view, this means that *M* controls the sensitivity of the voltage *E* with respect to the gating variable *n*.

**Fig. 19:**
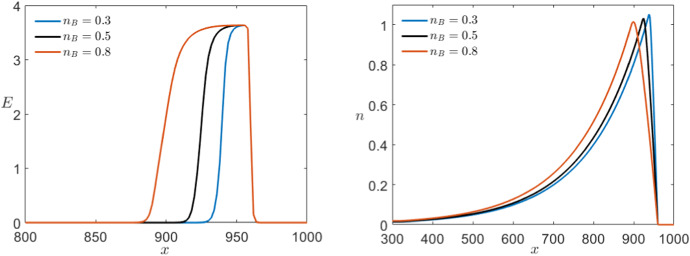
Comparison of the effect of multiple values of $n_B$ in a simulation of the Karma equations (2.13) with the same bump initial conditions shown in Fig. 11 for both variables *E* (left) and *n* (right) via a time shot at $t=550$ (Colour figure online)

On the other hand, we know that $0<n_B<1$ and, more precisely, we expect to normally encounter values lying between 0.3 and 0.8. In contrast to the previous case, if we allow $n_B>1$ then the unstable equilibrium changes stability and the system becomes bistable giving rise to completely different dynamics. Focusing on the range suggested by Karma, we find that the parameter $n_B$ determines the position of the wave back by controlling the speed of the slow subsystem. The higher $n_B<1$ the slower is the slow variable and therefore the longer is the depolarisation pulse (see Fig. 19).

**Fig. 20:**
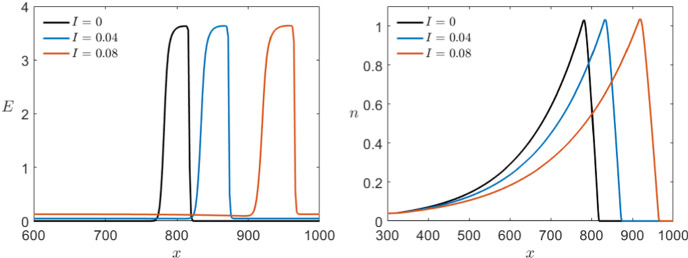
Comparison of the effect of multiple values of $0\le I<I_0$ in a simulation of the Karma equations (2.13) with the same bump initial conditions shown in Fig. 11 for both variables *E* (left) and *n* (right) via a time shot at $t=450$ (Colour figure online)

Last we can look at the effect of a small external current *I* in the Karma model. From the analysis of the ODE model in Sect. 3.1, we know that for $I=I_0\approx 0.087$ the system undergoes a saddle-node bifurcation so we cannot expect to have equivalent dynamics in the PDE case after crossing this point either. Nevertheless, we want to compare the system for $I<I_0$ since we expect to be able to extend the analysis above in this range. In Fig. 20, we see a time shot of the simulations for different values of *I*. At first sight, we see that again the wave speed is changed where the higher the external current the faster the propagation speed of the wave. We can also see that the base line is no longer 0 but slightly higher approaching the fold point as $I\rightarrow I_0$ as we would expect. For $I=0.08$, we start being able to see that by increasing the base line we also get a weak hyperpolarisation after the main pulse which we also would expect analytically due to the shape of the critical manifold.

**Fig. 21:**
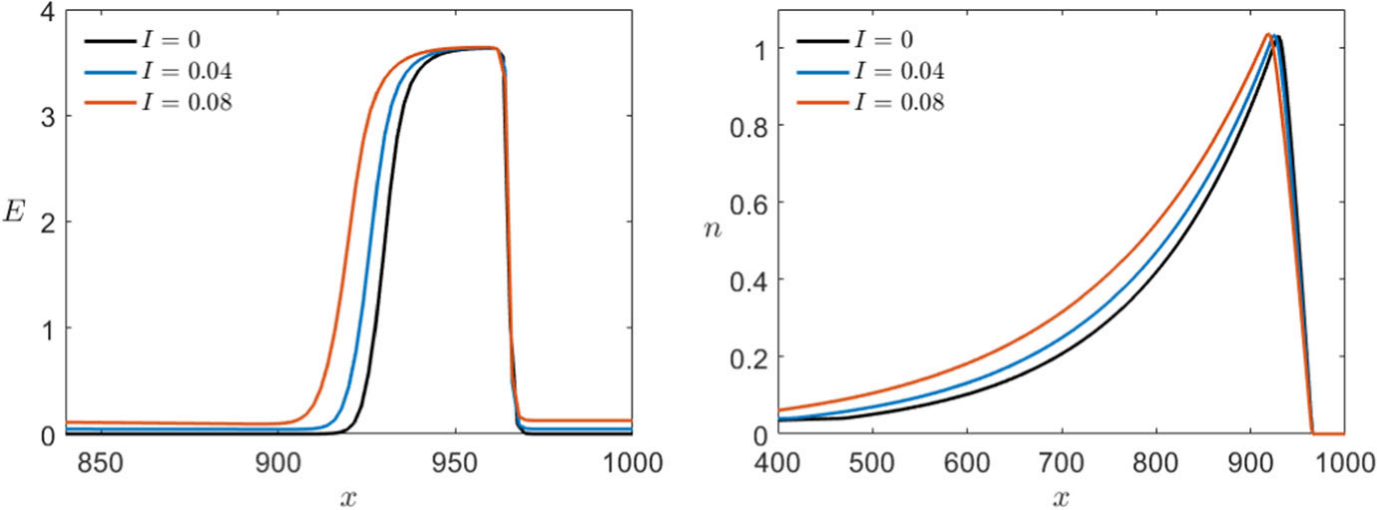
Comparison of the effect of multiple values of $0\le I<I_0$ on the profile of the pulse in a simulation of the Karma equations (2.13) with the same bump initial conditions shown in Fig. 11 for both variables *E* (left) and *n* (right) via shift of the pulses at $t=400$ along the *x*-axis such that the wave fronts coincide (Colour figure online)

Furthermore, we can shift the waves such that the wave fronts coincide and see that the wave profile is also affected by the external current (see Fig. 21). Although the effect is not as noticeable as the different wave speeds, we see that in addition to the higher base line we also have slightly longer pulses for higher incoming current.

## Discussion

We presented a systematic analysis and comparison of a polynomial version of the Karma model (Karma 1993, 1994) with the FHN model (FitzHugh 1955, 1960, 1961) motivated by applications to modelling excitable behaviour in cardiomyocytes with regard to individual cells as well as cell populations. We started by considering their pure ODE versions. In this setting, we noticed that Karma as well as FitzHugh–Nagumo present similar behaviours showing in both cases exactly three parameter regimes for the input current. When *I* is sufficiently small, the dynamics converge to a stable resting state while in the middle range of *I* both models oscillate following a globally stable limit cycle. Finally, when *I* is high enough any orbit converges to a stable equilibrium corresponding to a depolarised state. Nevertheless, although both systems are qualitatively similar, there are also some likely important differences when applying them to model cardiomyocytes. First, in the Karma model the re-polarisation is much slower than the depolarisation because of the sharpness of the critical manifold while in the FHN model both processes are of the same order. This difference is at the core of the prolonged wave form of the cardiac action potential compared to the classic narrow-shaped neuronal action potentials or spikes. This, in turn, has implications for electrical signal propagation in an excitable tissue. Also, for a high external input *I* the dynamics of the FHN model are still controlled by an “S”-shaped critical manifold, in other words, depending on the initial conditions it is possible to undergo a depolarisation and re-polarisation before converging to the stable state. In contrast to that, the Karma model does not allow any oscillation other than small fluctuations very close to the equilibrium given that in a reasonable regime for $\varepsilon$ the fixed point is a stable spiral. Yet, the biggest difference we see occurs when considering *I* as a dynamic variable instead of a parameter. In the extended phase space, we observe the high sensitivity to changes in *I* when the system is oscillatory and most importantly the cusp singularity that arises when the two folds collide. Because of these differences, it would be interesting in future work to look at the models with non-constant external current *I*. Interestingly, cusp singularities have been reported in a recent study performing bifurcation analysis of a human ventricular myocyte model with implications for efficient ways to create biological pacemakers (Ogawa and Doi 2016).

Next, we considered the spatially extended versions of the models and focused on travelling wave solutions in 1D without external current. This is motivated by our interest in modelling propagation of activity in populations of cardiomyocytes similar to Czeschik et al. (2015); Dang et al. (2018); Yakushenko et al. (2013). We start by analysing the 1D PDE in the singular limit $\varepsilon = 0$ in order to study the existence of travelling wave solutions. Here, using similar techniques as used for FHN in Guckenheimer and Kuehn (2009) in addition to singular perturbation theory, we have demonstrated the existence of travelling pulses originating and converging to a fixed resting state. The first difference we have found comparing the Karma model to FHN is the insensitivity of the wave speed to different values of the slow variable. Furthermore, in contrast to FitzHugh–Nagumo, the wave back in the Karma model starts at the fold point for large parameter ranges resulting as in the ODE system in much slower re-polarisation than the previous depolarisation. This is consistent with the characteristic speeds of the heart beat compared to the propagation of electrical signals in nerve tissues. All the analysis in this section has been restricted to $I=0$, therefore, as a future continuation of the work, it would be interesting to study if it is possible to extend the existence of travelling waves for $I>0$ and especially in the range where the ODE is oscillatory.

Finally, we performed numerical simulations of the 1D PDE Karma model varying model parameters. As we would expect, the propagation velocity does not depend on the parameters controlling the reactivity of the cells but only on the parameters defining the medium, namely the diffusion coefficient *D* and the background current *I*. On the other hand, while a change in *D* or *I* also affects the shape of the pulse we have observe that the main control over the shape is given by the reaction parameters $\varepsilon$, *M* and $n_B$. Since all these are based only on observations of different simulations, it would be another interesting avenue for future work to perform an even deeper analysis of the effect of the parameters on the travelling wave solutions.

## Theory for Multi-scale Systems

In this “Appendix”, we want to give an overview over the most important mathematical concepts and techniques used in this paper. We will start in Sect. A.1 by introducing some definitions and basic results related to the analysis of dynamical systems and, in particular, those ruled by multiple timescales (Jones 1995; Kuehn 2015; Wiggins 1994). Furthermore, we will present in Sect. A.2 the singular perturbation theory developed by Fenichel (1971, 1979) with its three key theorems.

### Definitions and Notation

First, some basic definitions for general dynamical systems.

#### Definition 1

For a set $S\subset {\mathbb {R}}^n$, a manifold $M\subset {\mathbb {R}}^n$ and a flow $\phi _t(\cdot )$ we have:

- *S* is called *invariant* under the flow $\phi _t(\cdot )$ if $\phi _t(S)\subset S$ for all $t\in {\mathbb {R}}$.
- *S* is called *positively invariant* if for all $p\in S$ it holds that $\phi _t(p)\in S$ for all $t\ge 0$.
- *S* is called *negatively invariant* if for all $p\in S$ it holds that $\phi _{-t}(p)\in S$ for all $t\ge 0$.
- *M* is called *locally invariant* under $\phi _t(\cdot )$ if for each $p\in \text {int}(M)$ there exists a time interval $I_p=(t_1,t_2)$ such that $0\in I_p$ and $\phi _t(p)\in M$ for all $t\in I_p$. In other words, the flow can only leave the manifold through its boundary.

We now continue with the systems showing multiple timescales. The setting we will be working with is a (*m*, *n*)-fast–slow system, this means we have an *m*-dimensional fast subsystem combined with *n* further variables moving at a slower timescale. The complete system is given by the $(m+n)$-dimensional system of differential equations

A.1 $$\begin{aligned} \begin{aligned} \varepsilon \frac{dx}{d\tau }&=\varepsilon {\dot{x}}=f(x,y,\varepsilon )\\ \frac{dy}{d\tau }&={\dot{y}}=g(x,y,\varepsilon ) \end{aligned} \end{aligned}$$

where $f\in C^r({\mathbb {R}}^{m+n+1},{\mathbb {R}}^m)$ and $g\in C^r({\mathbb {R}}^{m+n+1}, {\mathbb {R}}^n)$. For Eq. (A.1), the components of $x=x(t)\in {\mathbb {R}}^m$ are the *fast variables* and those of $y=y(t)\in {\mathbb {R}}^n$ are the *slow variables* of the system. The timescale separation is controlled by a small parameter $\varepsilon >0$, which provides the ratio between the *slow timescale* *t* to the *fast timescale* $\tau :=t/\varepsilon$. If we rewrite system on the fast timescale $\tau$, we obtain the *fast system* defined as

A.2 $$\begin{aligned} \begin{aligned} \frac{dx}{dt}&=x'=f(x,y,\varepsilon )\\ \frac{dy}{dt}&=y'=\varepsilon g(x,y,\varepsilon ). \end{aligned} \end{aligned}$$

In general, we are interested in the limit $\varepsilon \rightarrow 0$ for a given system, the so-called *singular limit*. Although the slow and fast system (A.1) and (A.2) are equivalent , their corresponding singular limit is not. Taking the singular limit of the slow system (A.1), we obtain the *reduced system* or *slow subsystem*

A.3 $$\begin{aligned} \begin{aligned} 0&=f(x,y,0)\\ {\dot{y}}&=g(x,y,0) \end{aligned} \end{aligned}$$

defining the so-called *reduced or slow flow*. On the other hand, the *layer problem* or *fast subsystem*

A.4 $$\begin{aligned} \begin{aligned} x'&=f(x,y,0)\\ y'&=0 \end{aligned} \end{aligned}$$

as limit for the fast system (A.2) defines the associated *fast flow*.

#### Definition 2

The algebraic constraint in (A.3) defines the *critical manifold*

$$\begin{aligned} C_0=\{(x,y)\in {\mathbb {R}}^{m+n}:f(x,y,0)=0\}. \end{aligned}$$

The points contained in the critical manifold correspond exactly to the equilibrium points of the fast subsystem.

#### Remark 11

The equation (A.3) defines the slow flow to be naturally restricted to the manifold $C_0$.

In this setting, we are able to decouple the fast and the slow dynamics in the system analysing them separately. Nevertheless, to obtain a global picture of the full system we need to combine the fast and the slow trajectories and we get the following definition.

#### Definition 3

A *candidate orbit* is the image of a homeomorphism $\gamma :(a,b)\rightarrow {\mathbb {R}}^{m+n}$ with $a<b$ and a partition $a=t_0<t_1<\dots <t_k=b$ for some $k\in {\mathbb {N}}^+$ such that

- the image $\gamma ((t_{i-1},t_i))$, $i\in \{1,\dots , k\}$ of each subinterval is a trajectory of either the fast or the slow subsystem
- the image $\gamma ((a,b))$ has an orientation that is consistent with the orientation of each trajectory $\gamma ((t_{i-1},t_i))$, $i\in \{1,\dots , k\}$

### Fenichel’s Theory

The following statements were first introduced by Fenichel (1971) and then applied to fast–slow systems (Fenichel 1979). Fenichel’s work consists of three main theorems posed in a very general setting. Since we will not need this generality, we will only present the important results already applied to fast–slow systems. We will not prove any of the statements in this section, for the proofs see Fenichel (1971, 1979); Kuehn (2015); Wiggins (1994).

To be able to understand the next theorems, we first need some additional definitions

#### Definition 4

A subset $S\subset C_0$ is called *normally hyperbolic* if the Jacobian with respect to the fast variables $D_xf(x^*,y^*,0)\in {\mathbb {R}}^{m\times m}$ has no eigenvalue with zero real part for all $(x^*,y^*,0)\in S$.

#### Remark 12

The definition shows that $S\subset C_0$ is normally hyperbolic if and only if for every $(x^*,y^*,0)\in S$ it holds that $x^*$ is a hyperbolic equilibrium of the fast subsystem for $y=y^*$, i.e. $x^*$ is a hyperbolic equilibrium of $x'=f(x,y^*,0)$.

#### Definition 5

Let $S\subset C_0$ be a normally hyperbolic set.

- *S* is called *attracting* if for all $(x^*,y^*,0)\in S$ every eigenvalue of $D_xf(x^*,y^*,0)$ has negative real part, i.e. for all $(x^*,y^*,0)\in S$ the corresponding equilibrium $x^*$ of the fast subsystem is stable for $y=y^*$.
- Similarly, *S* is called *repelling* if all eigenvalues have positive real part, i.e. the fixed points are unstable.
- If *S* is neither attracting nor repelling, it is called *of saddle type*.

#### Definition 6

The *Hausdorff distance* $d_H$ between to nonempty sets $V,W\subset {\mathbb {R}}^{k}$ is defined by

$$\begin{aligned} d_H(V,W):=\max \left\{ \sup _{v\in V} \text {dist}(v,W),~\sup _{w\in W}\text {dist}(w,V) \right\} \end{aligned}$$

where $\text {dist}(p,M):=\inf _{q\in M}||p-q||$ gives us the distance from a point $p\in {\mathbb {R}}^{k}$ to the set $M\subset {\mathbb {R}}^{k}$. In other words, the Hausdorff distance $d_H(V,W)$ defines the maximal distance between a random point in one set to the other set.

#### Theorem 10

(Fenichel’s first Theorem, fast–slow version) Let $S_0$ be a compact normally hyperbolic submanifold of the critical manifold $C_0$ of (A.1) and $f\in C^r({\mathbb {R}}^{m+n+1},{\mathbb {R}}^m)$, $g\in C^r({\mathbb {R}}^{m+n+1},{\mathbb {R}}^n)$ for $1\le r<\infty$. Then, for $\varepsilon >0$ sufficiently small it holds that

1. There exists a locally invariant manifold $S_\varepsilon$ diffeomorphic to $S_0$,
2. $S_\varepsilon$ has Hausdorff distance ${\mathscr {O}}(\varepsilon )$ from $S_0$,
3. The flow on $S_\varepsilon$ converges to the slow flow as $\varepsilon \rightarrow 0$,
4. $$S_\varepsilon $$ is $$C^r$$-smooth.

#### Definition 7

The manifold $S_\varepsilon$ obtained as conclusion of Theorem 10 is called a *slow manifold*.

#### Remark 13

$S_\varepsilon$ is usually not unique. Nevertheless, in regions lying at a fixed distance from $\partial S_\varepsilon$, all manifolds satisfying (F1)–(F4) lie at a Hausdorff distance ${\mathscr {O}}(e^{-K/\varepsilon })$ from each other for some positive $K\in {\mathscr {O}}(1)$. For this reason, a representative of the manifolds is often called “the” slow manifold since, in most cases, it is arbitrary which representative to choose.

By Theorem 10, we know that, starting with a fast–slow system in the singular limit, if we perturb the equations by taking $\varepsilon >0$ sufficiently small the structure and behaviour of the critical manifold do not disappear. Instead, any compact subset of the manifold perturbs continuously to a slow manifold $S_\varepsilon$. The perturbation does not only preserve the topological structure of the critical manifold but the flow on the slow manifold is also $\varepsilon$-close to the original slow flow.

#### Theorem 11

(Fenichel’s second Theorem) Given the setting as in Theorem 10, the statements (F1)–(F4) hold for the local stable and unstable manifolds if we replace $S_0$ and $S_\varepsilon$ by $W^{s,u}_{loc}(S_0)$ and $W^{s,u}_{loc}(S_\varepsilon )$ with

$$\begin{aligned} W^{s,u}_{loc}(S_0)=\bigcup _{p\in S_0}W^{s,u}_{loc}(p). \end{aligned}$$

In particular, $W^{s,u}_{loc}(S_\varepsilon )$ exist and furthermore $S_\varepsilon$ is normally hyperbolic and has the same stability properties with respect to the fast variables as $S_0$ (attracting, repelling or of saddle type).

Although the stable and unstable manifolds can only be defined for an equilibrium, with the help of Fenichel’s second Theorem we are able to generalise this notion to fast–slow systems with $\varepsilon$ small enough. The stable and unstable manifolds $W^{s,u}_{loc}(S_0)$ that result from the union of those of the individual fixed points of the fast subsystem are not lost by the perturbation but instead we find that the topological as well as the analytical properties in new manifolds $W^{s,u}_{loc}(S_\varepsilon )$ remain similar to those of the original ones.

#### Theorem 12

(Fenichel’s third Theorem) We start again with the same setting as in Theorem 10. Then, there exists a manifold ${\mathscr {F}}^u(p)$ for each $p\in S_0$ such that

- $$\bigcup _{p\in S_0}{\mathscr {F}}^u(p)=W^u_{loc}(S_0)$$,
- For $p\ne p'$ it holds that ${\mathscr {F}}^u(p)\cap {\mathscr {F}}^u(p')=\emptyset$,
- $$\phi _{-t}({\mathscr {F}}^u(p))\subseteq {\mathscr {F}}^u(\phi _{-t}(p))$$,
- ${\mathscr {F}}^u(p)$ is tangent to ${\mathscr {N}}^u_p$ at *p* with ${\mathscr {N}}_p^u$ the unstable component of the normal direction to $S_0$ (fast direction),
- There exist constants $C_u,\lambda _u>0$ such that if $q\in {\mathscr {F}}^u(p)$, then
  $$\begin{aligned} ||\phi _{-t}(p)-\phi _{-t}(q)||<C_ue^{-\lambda _ut} \end{aligned}$$
  for every $$t\ge 0$$,
- ${\mathscr {F}}^u(p)$ is $C^r$ with respect to the base point *p*.

The same conclusions (a)–(f) with the obvious modifications hold for the family of manifolds ${\mathscr {F}}^s(p)$, e.g. replace $-t$ by *t* in (c) so that $\phi _t({\mathscr {F}}^s(p))\subseteq {\mathscr {F}}^s(\phi _t(p))$. Furthermore, the foliation persists for $\varepsilon >0$ with all properties mentioned above and diffeomorphic to the foliation in the singular limit.

#### Definition 8

The manifolds ${\mathscr {F}}^{s,u}(p)$ are called the *stable/unstable fibres* through *p*.

The families ${\mathscr {F}}^{s,u}$ build decompositions of the stable/unstable manifolds by submanifolds characterised by initial conditions approaching each other in forward/backward time. Fenichel’s third theorem says that the asymptotic behaviour on the stable and unstable manifolds stays unchanged for positive $\varepsilon$.
